# Ultra-large supramolecular coordination cages composed of endohedral Archimedean and Platonic bodies

**DOI:** 10.1038/ncomms15268

**Published:** 2017-05-09

**Authors:** Kevin Byrne, Muhammad Zubair, Nianyong Zhu, Xiao-Ping Zhou, Daniel S. Fox, Hongzhou Zhang, Brendan Twamley, Matthew J. Lennox, Tina Düren, Wolfgang Schmitt

**Affiliations:** 1 School of Chemistry, University of Dublin, Trinity College, Dublin 2, Ireland; 2 CRANN Nanotechnology Institute & AMBER, Centre University of Dublin, Trinity College, Dublin 2, Ireland; 3 Institute of Molecular Functional Materials & Department of Chemistry, Hong Kong Baptist University, 224 Waterloo Road, Hong Kong, Hong Kong; 4 School of Physics University of Dublin, Trinity College, Dublin 2, Ireland; 5 Centre for Advanced Separations Engineering, Department of Chemical Engineering, University of Bath, Bath BA2 7AY, UK

## Abstract

Pioneered by Lehn, Cram, Peterson and Breslow, supramolecular chemistry concepts have evolved providing fundamental knowledge of the relationships between the structures and reactivities of organized molecules. A particular fascinating class of metallo-supramolecular molecules are hollow coordination cages that provide cavities of molecular dimensions promoting applications in diverse areas including catalysis, enzyme mimetics and material science. Here we report the synthesis of coordination cages with exceptional cross-sectional diameters that are composed of multiple sub-cages providing numerous distinctive binding sites through labile coordination solvent molecules. The building principles, involving Archimedean and Platonic bodies, renders these supramolecular keplerates as a class of cages whose composition and topological aspects compare to characteristics of edge-transitive {Cu_2_} MOFs with *A*_*3*_*X*_4_ stoichiometry. The nature of the cavities in these double-shell metal-organic polyhedra and their inner/outer binding sites provide perspectives for post-synthetic functionalizations, separations and catalysis. Transmission electron microscopy studies demonstrate that single molecules are experimentally accessible.

## Abstract (web summary)

Host–guest chemistry in hollow coordination cages can be exploited for a range of applications, but is often limited by inner cavity dimensions. Here, Schmitt and co-workers fabricate supramolecular keplerates that possess ultra-large cross-sectional diameters and are composed of multiple sub-cages.

Molecular coordination cages and metal-organic frameworks (MOFs) can be regarded as related metallo-supramolecular materials that consist of metal ions or polynuclear complexes linked through organic ligands to form extended networks or molecular species with defined cavities^1^,^2^,^3^,^4^. In the last decades, the former class of compound received significant scientific attention^5^,^6^,^7^,^8^,^9^,^10^,^11^,^12^,^13^,^14^,^15^,^16^. Preparative efforts were primarily directed towards the synthesis of new cage topologies with controllable inner cavities whose chemical, geometrical and electronic attributes can give rise to space-restricted properties that are exploitable in catalytic processes^5^,^6^,^17^,^18^, for separations^19^,^20^, drug delivery^21^ and sensing^22^,^23^. Most prominent synthetic approaches to functional capsules generally take advantage of kinetically stable building units with defined ligand-accessible coordination sites which direct the self-assembly into hollow structures, for example, in the presence of pyridine or imine ligands^5^,^6^,^7^,^24^. Other concepts to hollow molecular entities employ ‘reticular', topological considerations of inorganic and organic nodes which are generally applicable to MOFs^25^, and result in metal-organic polyhedra (MOP)^8^. In this context dinuclear {Cu_2_} acetate-based ‘paddlewheel' complexes represent versatile secondary building units (SBUs) with well-understood functional group compatibility^8^,^26^, but limitations that often arise from the insolubility of the resulting neutral MOP^27^.

Among the most intriguing developments in the field of supramolecular chemistry are examples that show how molecular cages can provide avenues to synthetic macromolecules with enzyme-type characteristics^28^,^29^,^30^,^31^, demonstrating how hollow local binding environments influence the transition states and ultimately performances of catalytic transformations. However, as new research strands are emerging and target guests are becoming more complex, achievements are hampered by a limited availability of capsules with large-diameter inner cavities whose dimensions, pore openings, surface properties and ligand characteristics allow the uptake and release of desired species at appropriate diffusion rates^32^,^33^,^34^,^35^,^36^. Representative large-diameter cages have been reported by Stang and Fujita and co-workers^32^,^33^,^34^,^36^. Furthermore, species with various distinctive inner binding sites that regulate intermolecular interactions between multiple reactants are expected to result in enhanced reaction rates and selectivities or may be applicable for the preparation of multifunctional supramolecular materials. Thus assembly principles that produce highly porous ‘super-cages' with endohedral, ‘onion'-type sub-shells or that are composed of multiple smaller sub-cages, are anticipated to provide a new class of functional molecular hosts^37^,^38^,^39^,^40^,^41^.

We demonstrate how the rotational flexibility of an extended, tri-functional ligand, 3,3′,3″-(benzene-1,3,5-triyltris(ethyne-2,1-diyl)tribenzoic acid (*m*-BTEB), can be exploited to prepare {Cu_2_}-based coordination cages with exceptionally large cross-sectional diameters and which are composed of outer- and inner-located Archimedean and Platonic bodies. The molecular species represent highly augmented MOP whose complexity and topological characteristics compare to key structural attributes of MOFs. While the conformational *anti, anti*-arrangement of two benzoate moieties of the *m*-BTEB ligand gives rise to a 120° binding geometry that promotes the formation of the outer shell, the rotational flexibility of the third alkyne-benzoate moiety promotes the formation of inner cages and allows the inner-located dinuclear {Cu_2_} complexes to adopt variable arrangements. The labile coordination sites of the SBUs facilitate the post-synthetic interaction with co-ligands that solubilize the nanoscopic molecular species or with smaller guest molecules. The molecules described here are among the largest carboxylate-stabilized cages reported. The studies are supported by X-ray crystallography, electron microscopy, dynamic light scattering (DLS) and vibrational, UV-vis, steady-state and time-resolved fluorescence spectroscopy.

## Results

### Synthesis and structural analysis

**Figure 1:**
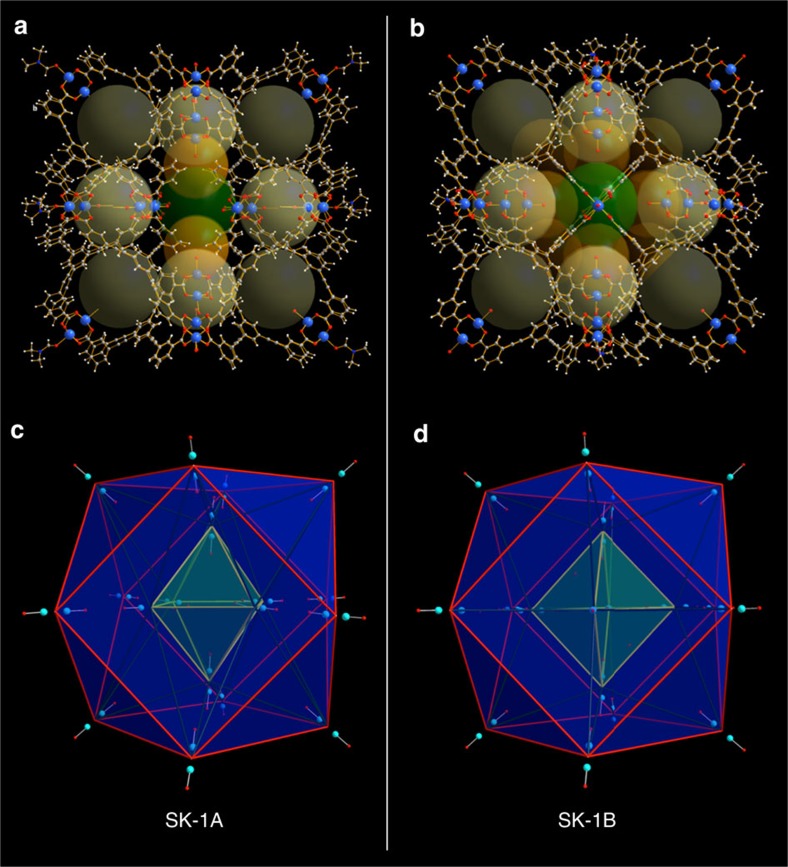
Structure of SK-1. (**a**,**b**) Structures of the SK-1A and SK-1B. (**c**,**d**) Endohedral arrangement of Archimedean and Platonic bodies in SK-1A and SK-1B; inner octahedra are rotated in their basal planes by ca. 45° in SK-1A and SK-1B, respectively.

**Figure 2:**
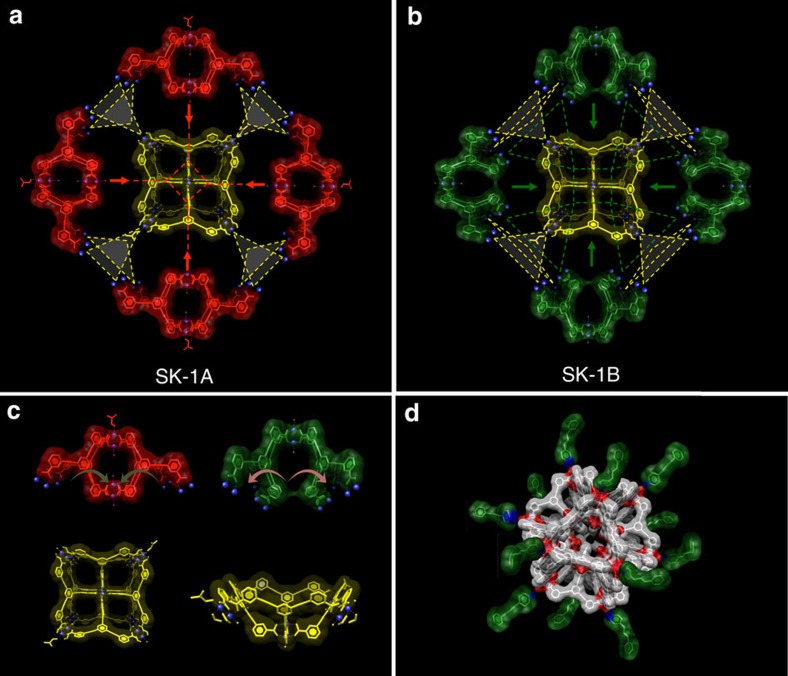
Sub-cages and building units of SK-1. (**a**,**b**) Building principles of SK-1A and SK-1B. Intersected lines indicate fusion points. Yellow lines give rise to the formation of square faces of the cuboctahedron (yellow units in **c**); Green and red lines indicate the position of the inner-located {Cu_2_} units that form part of the inner octahedron. In SK-1A only two {Cu_2_} units locate below square face (yellow units in **a**) leaving the remaining four faces open while in SK-1B all six inner {Cu_2_} units locate directly below square faces. (**c**) Structural motifs in SK-1A and SK-1B. Red: ‘Super-paddlewheel' cage that results from a *syn, syn*-binding mode of two benzoate functionalities of the *m*-BTEB ligand and whose guest binding ability is significantly determined by the inter-(Cu_2_} distance in the inner cavity; approximately co-planar arrangement of benzoate rings. Green: Cage motif that results from a *syn, anti*-binding mode of two benzoate functionalities of the *m*-BTEB ligand; approx. perpendicular arrangement of one benzoate ring with respect to the other outer two benzoate rings of the *m*-BTEB ligand; Yellow: ‘Capping' motif with square topology; one of the benzoate ligands of the *m*-BTEB moieties adopts an approximate perpendicular arrangement with respect to the other two benzoate rings. (**d**) Schematic representation of the possible functionalization using 4-(3-phenylpropyl)pyridine (PPP).

**Figure 3:**
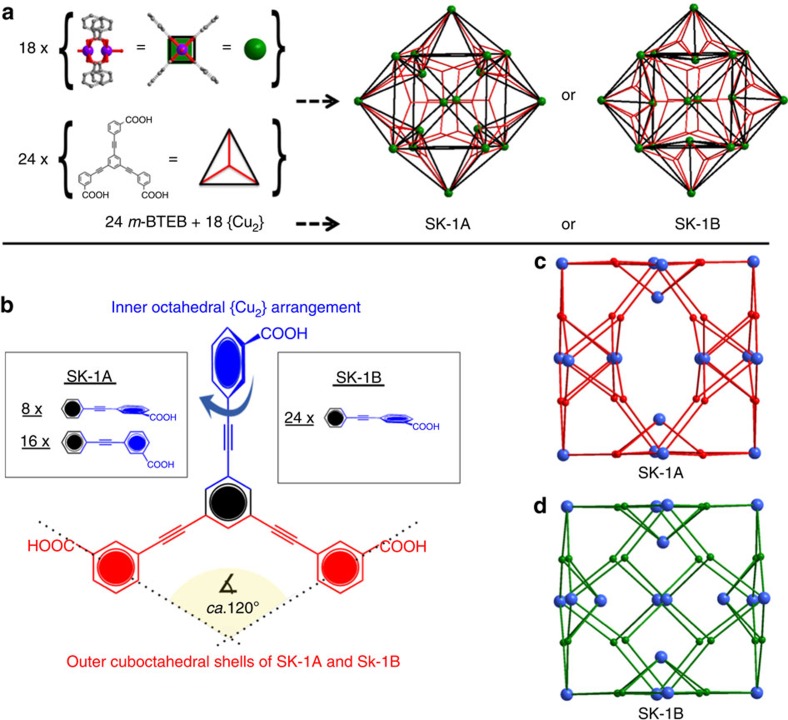
Ligand structure and benzoate conformations that promote the formation of SK-1. (**a**) Schematic representation of the formation of the coordination cages. (**b**) Arrangement of the benzoate moieties of the *m*-BTEB ligand in SK-1A and SK-1B. (**c**,**d**) Topological representations of SK-1A and SK-1B, respectively.

**Figure 4:**
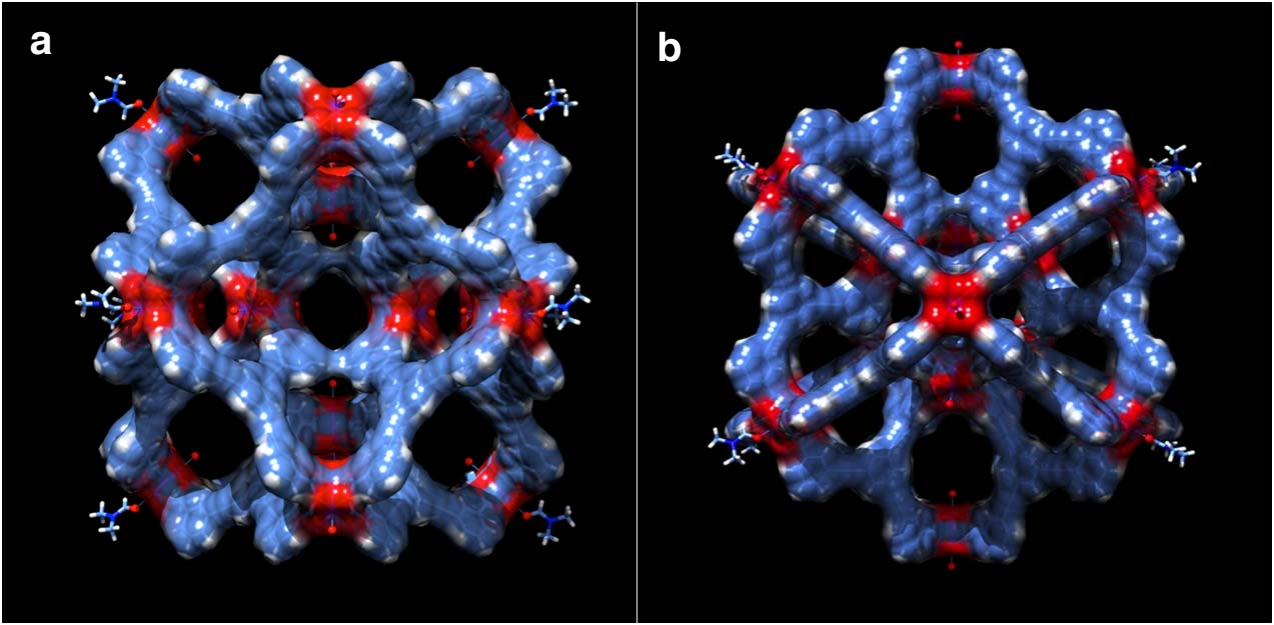
Space-filling representations of SK-1A. The molecular structure is shown from two different perspectives (**a**,**b**); Colour code, blue C, red O-donor coordination in the {Cu_2_} units, white H, dark blue N; coordinating solvent molecules are shown in non space-filling representations.

At initial stages of the presented work we were particularly interested in the rotational flexibility associated with the acetylene moiety of the *m*-BTEB ligand, which was expected to produce a number of isomeric coordination compounds^42^. This rotational freedom is a feature of extended ligands that incorporate acetylene moieties and distinguishes these linker-types from less-extended stericially restrained derivatives. While the trifunctional, triangular topology of the ligands gives rise to neutral 3,4-connected {Cu_2_}-based structures, one could expect that a possible 120° carboxylate binding arrangement that is typically associated with the *meta*-substitution could result in molecular, 0-periodic cages that may form next to extended MOF structures. Crystals of [Cu_36_(*m*-BTEB)_24_(H_2_O)_28_(DMF)_8_]·solv (SK-1) form reproducibly when *m*-BTEB and Cu(NO_3_)_2_·3H_2_O are reacted in dimethylformamide (DMF) at 100 °C for 5 days. While equimolar Cu(II):*m*-BTEB ratios favour the formation of merohedrally twinned crystals, higher Cu(II) concentrations promote the formation of blue rod-shaped crystals of SK-1. The characteristic blue colour of SK-1 allows a facile identification of the compound using an optical microscope and the crystals were manually separated from an insoluble co-precipitating green powder (Supplementary Fig. 1). Single-crystal diffraction experiments demonstrate that SK-1 crystallizes in the space group *C*2*/m* (No.12) in the monoclinic crystal system (Supplementary Table 1, Supplementary Note 1). The coordination compound is composed of molecular coordination cages resulting from the self-assembly involving 96 components (Figs 1, 2, 3, 4). The molecular cages reveal endohedral, onion-type arrangements and have the empirical formulae [Cu_36_(*m*-BTEB)_24_(H_2_O)_28_(DMF)_8_] (Figs 1 and 3). The SBUs of the coordination cages are dinuclear {Cu_2_} ‘paddlewheel' complexes in which O-donor atoms of *syn, syn*-bidentate carboxylate groups of the deprotonated, organic ligands provide the basal plane of the square pyramidal coordination polyhedra; O-donor atoms of coordinating H_2_O and DMF molecules bind in the apical positions. The observed structural characteristics of the {Cu_2_} units closely compare to those observed in other MOPs or MOFs^10^,^26^,^42^,^43^,^44^. The Cu–O bond lengths of the outer located {Cu_2_} units in SK-1 involving the carboxylate functionalities vary between 1.939(5) and 1.985(6) Å while Cu–O bond distances of the coordinating solvent molecules range between 2.106(5) and 2.166(7) Å.

The topology of the outer-shell of the cages is best described as a cuboctahedron (cuo), whereby the centres of the Cu–Cu axes in the {Cu_2_} units represent the nodal vertices (Figs 1 and 3). The resulting outer-sphere is characterized by largest cross-sectional diameters of ca. 48–50 Å depending on the direction of view (distance between O-atoms of opposite-located, coordination solvent molecules). The outer-shell structure results from an anti-alignment of adjacent benzoate moieties of the *m*-BTEB ligands (red-coloured ligand representation in Fig. 3b) whereby both benzoate rings adopt approximately co-planar arrangement with respect to the central aromatic ring. The third benzoate moiety of the *m*-BTEB ligand links the outer-shell to six further dinuclear {Cu_2_} units which are located in the inner cavity. The six {Cu_2_} SBUs in the inside of the sphere are disordered over 18 different positions; each inner phenyl ring of the *m*-BTEB ligand adopts three possible different orientations. Owing to this rotational flexibility of the acetylene moieties different octahedral {Cu_2_} arrangements within the cuboctahedral shell are observed whereby two structurally distinguishable, isomeric forms SK-1A and SK-1B are possible. SK-1A results when some benzoate moieties that point to the inside of the cage adopt approximately a co-planar arrangement with respect to the central ring. The building units of this species can be represented as four ‘super-paddlewheel' cages^45^ that assemble with two capping units as schematically represented in Fig. 2a,c (yellow and red subunits). This assembly results in an octahedral arrangement of the six inner {Cu_2_} units in which two SBUs locate below the square faces and four locate directly below vertices of the outer cuboctahedral shell. This inner arrangement leaves the four square faces open resulting in large pore openings that can facilitate the uptake or release of guests. The second possible stereoisomer SK-1B results when all benzoate moieties which point to the inside are arranged approximately perpendicular to the central aromatic ring. This conformation results in an octahedral arrangement of the inner 6 {Cu_2_} units that locate below the centres of the square faces of the cuboctahedral polygon. The formation of SK-1B can schematically be visualized considering two capping units with square topology and four ‘open'-subunits as represented in Fig. 2b,c (yellow and green subunits). The formal assembly of the latter ‘open'-subunits gives rise to the remaining four square faces of the cuboctahedral polygon at their fusion points. Thus, the key difference between SK-1A and SK-1B results from the orientation of the inner octahedral polygons, which are rotated in their basal planes by ca. 45°, with respect to each other. Both cages in SK-1 are characterized by an endohedral arrangement of Archimedean (cuboctahedral) and Platonic (octahedral) bodies; thus we classify these molecular species as supramolecular keplerates (SK)^46^.

This endohedral arrangement in SK-1 results in various, defined, interconnected void spaces with multiple binding sites and thus the structures and potential binding abilities are rather distinctive to those of large, single-volume cages. Indeed, the structures presented here resemble structural characteristics of extended MOFs and marry attributes of large-diameter cages with the presence of multiple small cages that can give rise to enhanced binding abilities. A topological consideration of the molecules and their empirical composition, {Cu_2_}_18_(*m*-BTEB)_24_={Cu_2_}_3_(*m*-BTEB)_4_, underpins their relationship to neutral ‘*A*_*3*_*X*_*4*_'-stoichiometric MOFs that assemble from SBUs with square and triangular nodes to form, for instance the edge-transitive default topologies that can be described by the RCSR symbols tbo and pto (for example, found in MOF-14 and HKUST-1)^43^,^44^. An enumeration of this special class of (4,3)-connected nets in which each 4-connected node is connected to four 3-connected nodes has been given by Wells^47^. Considering these topological aspects SK-1A and -B can be described as molecular structures with (3,4,4)-sub-connectivity, thus 3-nodal sub-structures containing two topologically distinguishable 4-connected nodes. The structural differences of SK-1A and -B are represented in unique sets of point symbols 4^6^(4.6^2^.8^3^)(4^2^.6) and (4^2^.6^2^.8^2^)(4^4^.6^2^)(4^2^.6), respectively (Fig. 3c,d). The different point symbols result from the outer and inner {Cu_2_} SBUs and further relate to the observed tilting between the central and outer phenyl rings of the *m*-BTEB ligand. Considering these structural attributes, both cages may also be classified as 0-periodic portions of {Cu_2_} paddlewheel-based MOFs.

Based on the refinement of the crystal structure and relative atom occupancies it is clear that SK-1A is the dominant structural form in SK-1 (Supplementary Movie 1, Supplementary Data 1) However based on the diffraction data and refined atom positions, the existence of SK-1B is structurally feasible and cannot be ruled out.

### Possible binding sites and void volumes in SK-1

A significant feature of {Cu_2_}-based MOFs results from the lability of the terminal coordination solvent molecules of the SBUs which can facilitate guest binding. Particularly, symmetric structures with linearly arranged {Cu_2_} SBUs can provide ‘molecular traps' for suitably sized molecules whose donor atoms facilitate the bridging between the two active coordination sites of adjacent SBUs^26^,^27^,^42^. In this context, the observed symmetric arrangement of the six {Cu_2_} units whose labile solvent molecules locate linearly opposite to each other and whose molecular axes point towards a single focal point is expected to be ideal to facilitate guest binding. The slightly tetragonally distorted octahedral inner arrangements in SK-1A are characterized by {Cu–Cu} distances of 16.7, 15.9 and 15.6 Å, and 16.6, 16.4 and 15.4 Å of opposite located {Cu_2_} units. Further binding sites are provided by {Cu_2_} units of the outer shell whose labile solvent sites point to the inside of the molecular entities. Noteworthy are the ‘super-paddlewheel' units in SK-1A which have previously been employed to trap suitably sized guests (for example, CO_2_ gas molecules, pyrazine or pyridine derivatives) between opposite located {Cu_2_} units^26^,^27^,^42^. The observed Cu–Cu distances in these sub-units in SK-1A vary between ca. 9.1 and 9.2 Å. The cages represent highly augmented species with exceptionally large void spaces and nanoscopic openings as visualized by the space-filling representations in Fig. 4 and Supplementary Fig. 2. Bulk crystals of SK-1 rapidly lose crystallinity upon desolvation, and the associated structural changes hamper gas storage measurements. Computational BET studies to evaluate the potential porosity and surface areas of the compound were conducted considering the structure of SK-1A. The adsorption of N_2_ at 77 K, modelled using grand canonical Monte Carlo simulations, gives rise to a type-I isotherm and a BET surface area of >4,100 m^2^ g^−1^. The calculated pore volume is 1.63 cm^3^g^−1^ while the pore-size distribution gives defined pores whose diameters range between ca. 6 and 16 Å (Supplementary Fig. 3, Supplementary Note 2). Based on the crystal data, the largest pore openings involving the outer square and triangular faces are represented by max. cross-sectional distances of ca. 9.8 Å (C–C atom distance between opposite phenyl rings) and 6.8 Å (C–C atom distance between adjacent phenyl rings), respectively. In SK-1A the four square openings allow two orthogonal channels to extend through the entire diameter of the cage. This structural feature of SK-1A provides facile access to the central cavity and is expected to promote uptake or release of guest molecules at high diffusion rates. One should note that where {Cu_2_} units locate below the centre of the square faces, four smaller windows provide accessibility to the inner sphere. In addition, triangular faces afford accessible windows to the binding sites associated with the sub-cages or ‘molecular traps' that make up SK-1. The individual coordination cages in the crystal structure form distorted hexagonal arrangements within the crystallographic (001) plane and pack directly on top of each other in the direction of the crystallographic *c*-axis (Supplementary Fig. 4). Despite this relatively dense packing of the molecular entities the compound is characterized by an exceptionally large solvent accessible void volume that accounts for more of than 70% of the unit cell volume. Considering the packing arrangement, a significant part of the void space that can give rise to porosity can be attributed to the structure of the molecular species.

### Solution behaviour and electron microscopy

**Figure 5:**
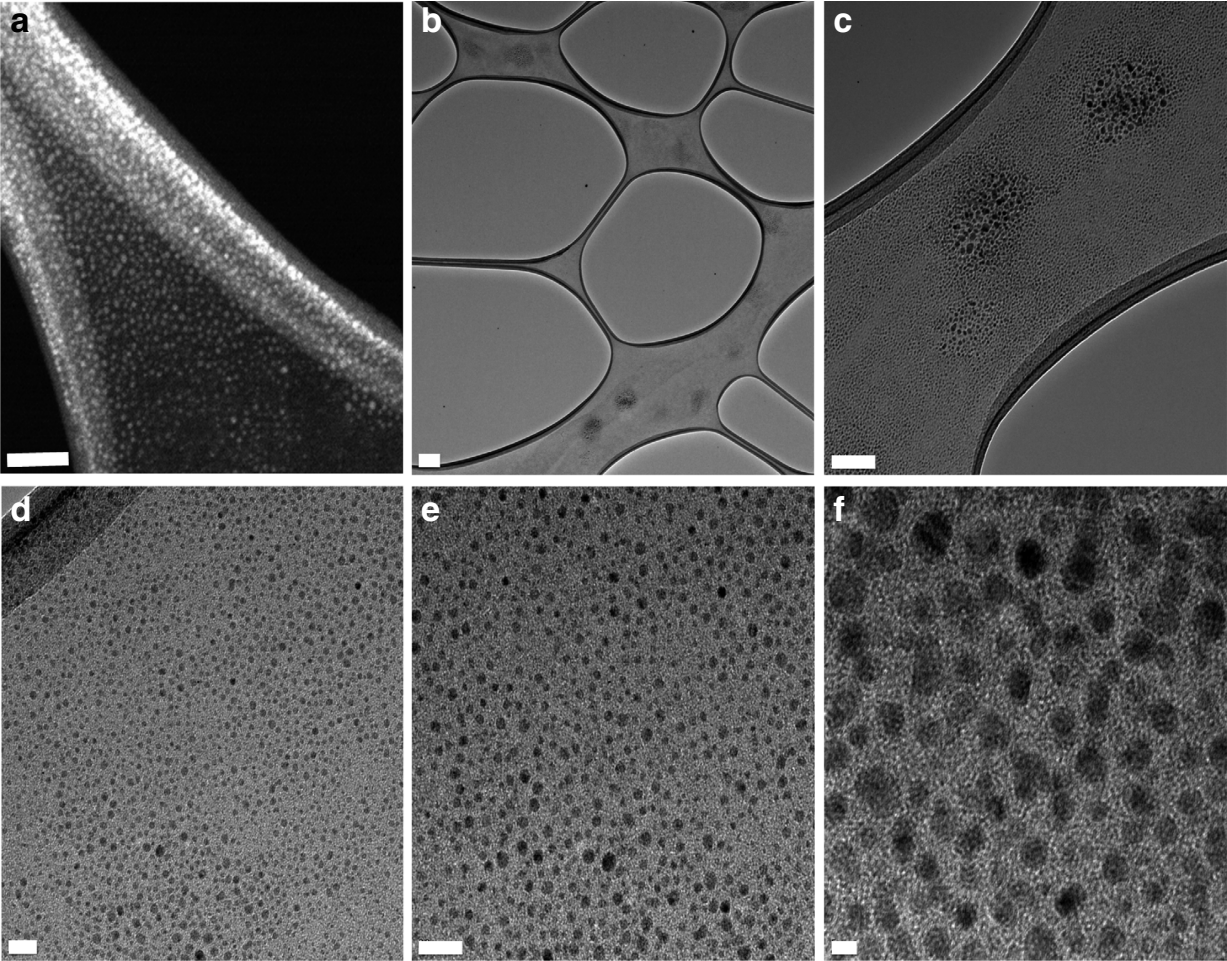
Electron microscopy of SK-1 whereby SK-1/PPP/CHCl_3_ solutions that were drop-casted onto TEM grids. (**a**) Scanning transmission electron micrograph (high angle annular dark field STEM); (**b**–**f**) Transmission electron micrographs at increasing magnification (bright field TEM). The scale bars equal 100 nm (**a**), 200 nm (**b**), 100 nm (**c**), 20 nm (**d**,**e**), 5 nm (**f**).

**Figure 6:**
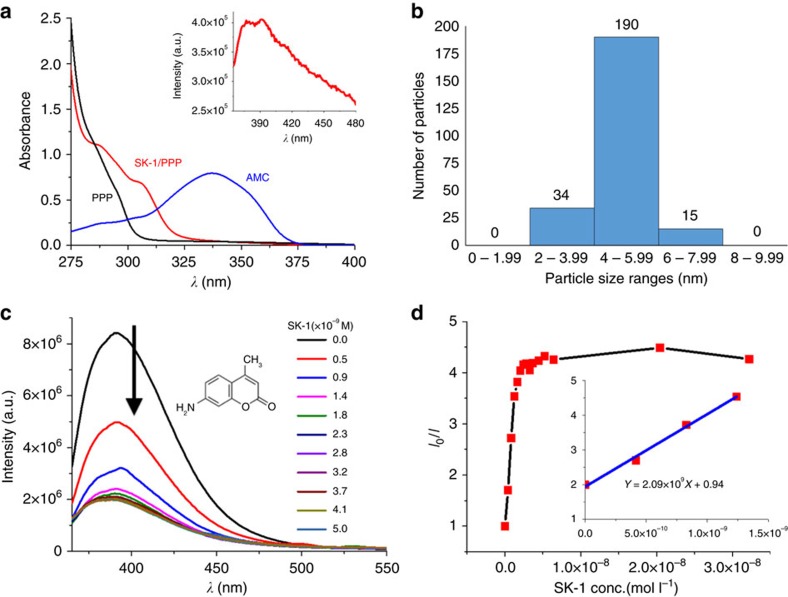
Particle-size distribution and solution behaviour of SK-1 cages in CHCl_3_. (**a**) UV-vis spectra of PPP in CHCl_3_ ([PPP]∼1 × 10^−5^ M, black line), SK-1/PPP in CHCl_3_ ([SK-1]∼2 × 10^−5^ M, [PPP]∼7.5 × 10^−4^ M, red line) and 7-amino-4-methylcoumarin (AMC) in CHCl_3_ ([AMC]∼1 × 10^−5^ M, blue); (**a**) *inset* Fluorescence spectrum of SK-1/PPP in CHCl_3_ ([SK-1]∼2 × 10^−6^ M, [PPP]∼7.5 × 10^−5^ M, *λ*_ex_=338 nm); (**b**) Particle-size distribution (measured from Fig. 5e); (**c**) Fluorescence titrations characterizing the host–guest interactions between 7-amino-4-methylcoumarin (AMC) and the coordination cages (*λ*_em_max_ ca. 391 nm; *λ*_ex_=338 nm); Fluorescence quenching when aliquots of a SK-1/PPP solution in CHCl_3_ ([SK-1]∼2 × 10^−7^ M, [PPP]∼7.5 × 10^−6^ M) are added to an AMC solution in CHCl_3_ (1 × 10^−7^ M). (**d**) Stern–Volmer plots (*λ*_em_=391 nm; *λ*_ex_=338 nm) that are characteristic for static quenching and indicating binding (see inset) between the coordination cages and AMC.

Charge-neutral {Cu_2_}-based coordination cages are known to display low solubilities which hamper their use in homogeneous host–guest systems^27^,^45^. Indeed, also SK-1 is insoluble in most common polar and non-polar solvents. Ligand-modification and post-synthetic functionalizations using pyridine and pyridine derivatives are known strategies to influence the solubility of {Cu_2_}-based coordination cages and cluster species that possess labile coordination sites^48^,^49^,^50^. One observes, when long-chain, hydrophobic pyridines are introduced, crystals of SK-1 slowly dissolve in chloroform, dimethylformamide or toluene. The pyridines are expected to have a high affinity to interact with coordinating solvent molecules of the {Cu_2_} units and as such are generally expected to exchange at the {Cu_2_} binding sites, break-up the crystal packing structure and solubilize the molecular entities (Fig. 2d) which were characterized using electron and atomic force microscopy (AFM) (Fig. 5 and Supplementary Figs 5–8), light scattering and spectroscopic techniques (Fig. 6 and Supplementary Figs 9–11). The dissolution of SK-1 can be monitored using UV-vis spectroscopy (Fig. 6) whereby signals that arise from π–π* and n–π* transitions of the *m*-BTEB ligand can be traced. Upon dissolving individual crystals of SK-1 using 4-(3-phenylpropyl)pyridine (PPP) in CHCl_3_, weak bands emerge at 286 and 307 nm next to a strong signal whose absorbance maximum is reached below 260 nm that predominantly arises from the excess of PPP in solution (Fig. 6a). DLS experiments using SK-1/PPP solutions in CHCl_3_ give rise to a sharp signal which can be indicative of a well-defined, mono-disperse molecular species whose dimensions agree well with the crystallographic model (Supplementary Fig. 9a). Electron micrographs of these SK-1/PPP/CHCl_3_ solutions that were drop-casted on transmission electron microscopy (TEM) grids identify uniform, monodisperse particles whose size agrees well with a cross-sectional diameter of ca. 6 nm and is consistent with the structural model in which PPP ligands bind to the molecular species. Micrographs recorded at various magnifications (Fig. 5a–f) and the associated size distribution (Fig. 6b) visualize the clean and homogeneous nature of the deposition from solution and confirm that single molecular species of the cages are experimentally accessible. Energy dispersive X-ray (EDX) mapping confirms the copper content in the species (Supplementary Fig. 6e, *inset*) and EDX analysis gives rise to a C/Cu ratio of ca. 5.6 suggesting that ca. 20 PPP molecules interact with the individual capsular entities (Supplementary Fig. 12b). While it can be expected that the PPP molecules primarily attach at the periphery of the cages, it is also feasible that some of these pyridine derivatives enter the cages. The analysis is further substantiated by Infra-Red (IR) spectra of as-prepared crystals of SK-1 and surface-deposited molecules from SK-1/PPP/CHCl_3_ solutions. The resulting IR spectra of SK-1 are characterized by vibrational signals at 1,594 and 1,430 cm^−1^ corresponding to the asymmetric and symmetric carboxylate stretches; their positions (Δν=ν_a_−ν_s_=164 cm^−1^) agree with the observed *syn-syn* bidentate bridging mode of the benzoate functionalities of the *m*-BTEB ligands in dinuclear SBUs^42^. Depositions that result from the evaporation of SK-1/PPP/CHCl_3_ solutions give rise to IR spectra that confirm the presence of PPP molecules and spectral signatures that closely match those of the parent bulk, crystalline material of SK-1 (Supplementary Fig. 10).

Possible interactions between the solubilized cages and secondary small, functionalizing molecules that can potentially interact with the cages were evaluated in preliminary steady-state and time-resolved fluorescence experiments using 7-amino-4-methylcoumarin (AMC). It is observed that the fluorescence emission maximum of AMC/CHCl_3_ solutions ([AMC]=1 × 10^−7^ M, *λ*_em_ ca. 391 nm; *λ*_ex_=338 nm) is efficiently quenched when aliquots of SK-1/PPP ([SK-1]≈2 × 10^−7^ M in CHCl_3_) are added, leaving the lower intensity emission bands at 369 and 384 nm which originate from SK-1 (Fig. 6a, *inset*). This strong fluorescence quenching effect that possibly indicates that the fluorophore molecules bind to the cage species results in superimposable fluorescence spectra at an AMC/cage mole ratio of above 30:1 (Fig. 6c).

The interactions between AMC and the cage molecules and the kinetics of this intermolecular quenching process were evaluated by Stern–Volmer analyses. The *I*_0_/*I* versus [SK-1] plots as shown in Fig. 6d are characteristic for static quenching and the high K_Stern−Volmer_ constants (Fig. 6d, inset) are indicative of strong binding between potential host and guest molecules in solution. Corresponding fluorescence lifetime measurements involving additions of SK-1/PPP to an AMC solution in CHCl_3_ give typical mono-exponential decays in the nanosecond range. The observed lifetimes (Supplementary Fig. 13) do not depend on the relative quencher/fluorophore ratio, which further supports the static interactions between host and guest molecules in the ground state. Consecutive TEM analyses of the resulting SK-1/PPP/AMC/CHCl_3_ solutions that were drop-casted on grids give rise to mono-dispersed species (similar to those of previously discussed TEM analyses) confirming that the molecular cages maintain their integrity upon guest interaction (Supplementary Fig. 11e). Further, crystals of SK-1 when dispersed in an AMC/CHCl_3_ solution also adsorb AMC. During the adsorption the solid material of SK-1 undergoes a colour change to darker blue/green. The adsorption is accompanied by a decrease of fluorescence intensity and the IR analysis confirms the up-take of the coumarin derivative (Supplementary Fig. 14).

## Discussion

In summary, we report a synthetic approach to spherical coordination cages whose cross-sectional diameters are among the largest of crystallographically characterized synthetic, hollow supramolecular species. The tri-functional organic ligand promotes the formation of SK that can be solubilized and potentially functionalized through coordinative interactions at the labile coordination sites of the {Cu_2_} units. The coordination cages may be regarded as endohedral supramolecular cages that are composed of multiple smaller sub-cages providing numerous binding sites. The molecular species are composed of defined, various-sized cavities, contain different pore/channel openings and their topological features are comparable to those of ‘paddlewheel'-based MOFs with *A*_*3*_*X*_4_ composition. Correspondingly to endohedral Pd(II)/Pt(II)-based coordination cages^37^,^38^,^39^,^40^,^41^, the structural complexity distinguishes these SK from large-diameter, single-shell MOP and provides a new perspective to supramolecular host–guest approaches. Particularly noteworthy are the oppositely located labile coordination sites of the {Cu_2_} units that can facilitate guest binding and which are known to act as molecular traps^26^. Initial photophysical studies suggest that these sites, together with the intricate molecular structures give rise to guest-binding. Electron microscopy studies clearly demonstrate that the nanoscopic species are accessible on a single-molecule level. The structural characteristics of SK-1 are expected to provide possibilities for facile functionalization at the periphery, for complex separations and for catalytic transformations utilizing the different cavities of the sub-cages. Future research will investigate these aspects. Further, we will investigate the host–guest interactions/structures in more detail and focus on resolving, discrete atom conformations in the central cavity of the cages.

## Methods

### Synthesis of *m*-BTEB

*m*-BTEB was synthesized in a four-step synthesis according a modified, previously described synthetic procedure for the isomeric compound, 4,4′,4″-(benzene-1,3,5-triyltris(ethyne-2,1-diyl)tribenzoic acid (Supplementary Methods, Supplementary Fig. 15). ^1^H NMR (400 MHz, DMSO-*d*_6_) δH/ppm: 8.11 (3H, s, Ar–H), 7.94 (3H, d, *J*=7.70, Ar–H), 7.80 (3H, s, Ar–H), 7.62 (3H, d, *J*=7.54, Ar–H), 7.43 (3H, t, *J*=7.62, Ar–H). ^13^C NMR (150 MHz, DMSO-*d*_6_) δC/ppm: 166.4, 135.4, 134.2, 132.2, 131.5, 129.8, 129.2, 123.4, 122.0, 89.9, 88.1. HR-MS (ESI): m/z Calculated for C_33_H_17_O_6_ [M-H]^−^: 509.1025; Found: 509.1027. Overall yield ca. 20%.

### Synthesis of SK-1

7 × 10^−3^ g (0.029 mmol) of Cu(NO_3_)_2_.3H_2_O in 0.6 ml DMF was added to a dispersion of 1.5 × 10^−2^ g (0.029 mmol) of *m*-BTEB in 0.6 ml DMF. The reaction vial (1.5 ml capacity) was closed and heated at 100 °C for 5 days. The resulting crystals were separated manually, washed with DMF and kept under DMF. Estimated yield ca. 14% based on Cu(NO_3_)_2_.3H_2_O. IR (neat, cm^−1^): 3,417 (br, w), 2,929 (w), 1,652 (st), 1,626 (m), 1,594 (w), 1,496 (w), 1,430 (m), 1,386 (st), 1,254 (m), 1,091 (st), 1,062 (w), 986 (w), 920 (w), 879 (w), 765 (st), 686 (w), 659 (s), 533 (w), 486 (st); Elemental/EDX analyses see Supplementary Fig. 12a.

### Further characterizations

TEM and scanning transmission electron microscopy experiments were carried out using a CHCl_3_ solution (2 ml) containing SK-1 (ca. 1 × 10^–3^ g) and excess 4-(3-phenylpropyl)pyridine (PPP, ca. 100 μl). Ca. 0.15 ml of the former solution was drop-casted onto a carbon-coated copper grids and which were then dried overnight. The samples were imaged using a Titan field emission transmission electron microscope operating at an accelerating voltage of 80 kV or 300 kV. For EDX spectroscopy analyses, crystals of SK-1 were deposited on Si/SiO_2_ wafers and an SK-1/PPP/CHCl_3_ solution was drop-casted onto a Si_4_N_3_ grid; both samples were analysed using a Zeiss Ultra Plus scanning electron microscope. For AFM analyses, samples (0.1 mg SK-1, 0.05 ml PPP, 1 ml CHCl_3_) were dropcasted on HOPG and imaged using an Asylum Research AFM instrument. DLS experiments were conducted using a CHCl_3_ solution (3 ml) which contained SK-1 (ca. 1 × 10^–3^ g) and excess PPP (ca. 100 μl). The studies were performed using a Malvern Zeta Sizer Nano ZS analyser. For single crystal and powder X-ray diffraction studies, crystals of SK-1 were mounted in a capillary containing a small amount of DMF. Data were collected in capillaries using DMF solvent on a Bruker APEX II DUO CCD diffractometer equipped with a I*μ*S CuKα microfocus tube (wavelength 1.54184 Å). See Supplementary Fig. 1f. For photophysical studies, stock solutions containing SK-1, PPP and AMC were prepared using HPLC grade CHCl_3_. UV-vis absorbance spectra were recorded in 1 cm quartz cuvettes using a PerkinElmer Lambda 1050 UV/vis/NIR spectrophotometer. Baseline corrections were applied to all spectra. Emission spectra and fluorescence lifetime measurements were performed using a Fluoromax 4 (Horiba-Jobin-Yvon) spectrometer and a Fluorolog, TCSPC system (Horiba-Jobin-Yvon) equipped with a 295 nm excitation source (nanoLED-295). Fluorescence decay curves were fitted using the Data Station DAS6 software package. Mass spectra were recorded using a Waters Alliance Micromass LCT classic spectrometer (Supplementary Figs 16 and 17).

### Data availability

The data supporting the findings of this study are available within the article and its Supplementary Information. The X-ray crystallographic coordinates for the structure reported in this study have been deposited at the Cambridge Crystallographic Data Centre (CCDC) under deposition number 1491341. These data can be obtained free of charge from The CCDC via http://www.ccdc.cam.ac.uk/data_request/cif. Crystallographic data for SK-1 are shown in Supplementary Table 1.

## Additional information

**How to cite this article:** Byrne, K. *et al*. Ultra-large supramolecular coordination cages composed of endohedral Archimedean and Platonic bodies. *Nat. Commun.* **8,** 15268 doi: 10.1038/ncomms15268 (2017).

**Publisher's note**: Springer Nature remains neutral with regard to jurisdictional claims in published maps and institutional affiliations.

## Supplementary Material

Supplementary Information Supplementary Figures, Supplementary Table, Supplementary Notes, Supplementary Methods and Supplementary References

Supplementary Data 1 Structural coordinates of SK-1A (res-file in which disordered atom positions have been deleted for clarity).

Supplementary Movie 1 Structure of SK-1A in ball-stick and space-filling representations. Colour code: blue Cu, red O, grey C, darker blue N, white H). A simplified topological representation highlights the endohedral arrangement of archimedean and platonic bodies.
