# Supplementary material for: Ultra-large supramolecular coordination cages composed of endohedral Archimedean and Platonic bodies
Source: Nat Commun. 2017 May 9;8:15268. doi: 10.1038/ncomms15268 (PMC5436142; doi:10.1038/ncomms15268)
Supplement: Supplementary Information — Supplementary Figures, Supplementary Table, Supplementary Notes, Supplementary Methods and Supplementary References [file ncomms15268-s1.pdf]

Supplementary Figures & Tables

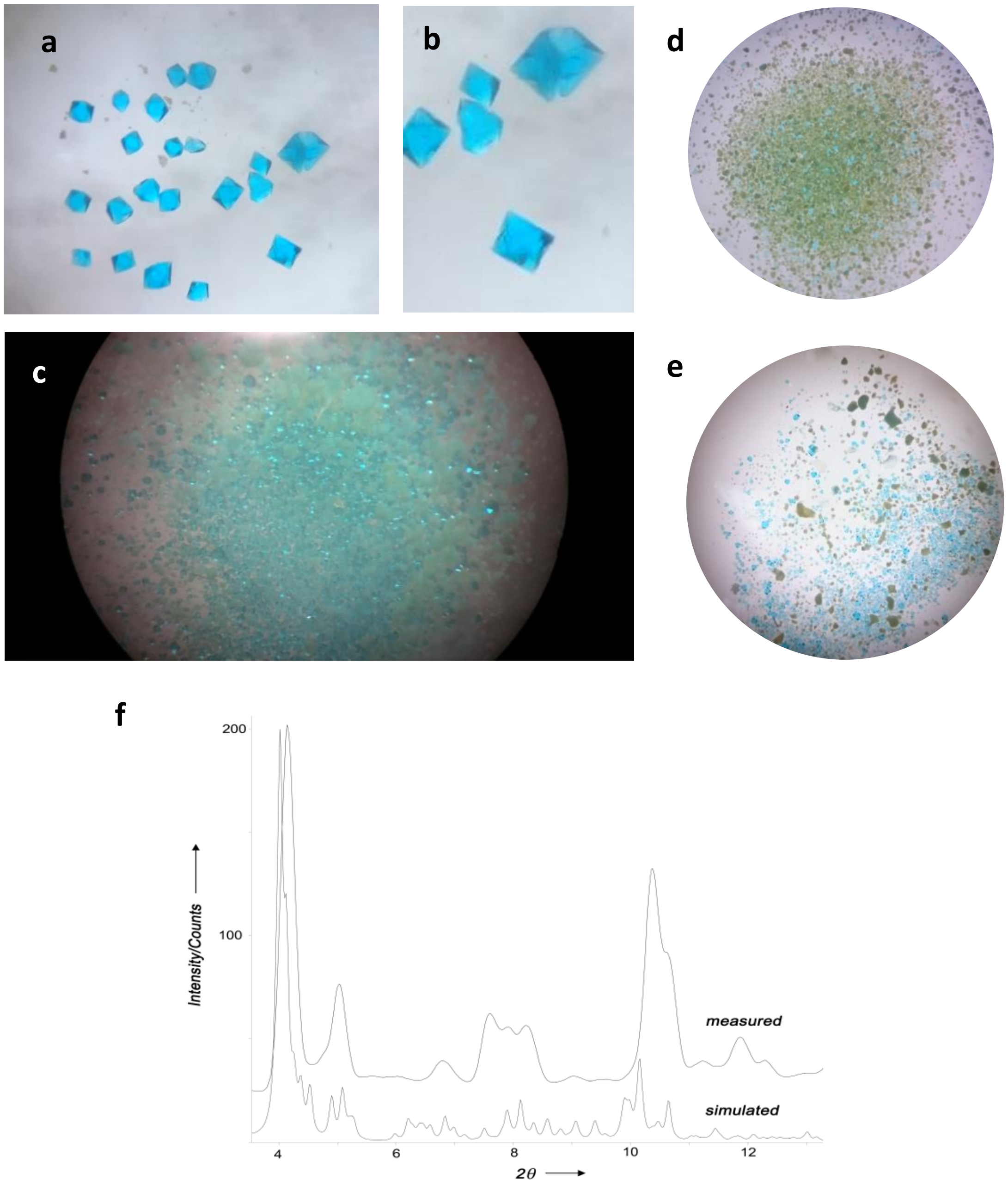

Supplementary Figure 1 | **Sample of SK-1**; **a-c** purified by manual separation of the crystals from the product mixture; **d & e** typical co-precipitation of **SK-1** with a green unidentified material. **f** Powder X-ray diffraction pattern of the bulk sample of SK-1. Measured (top) and simulated pattern (bottom, simulated based on single crystal X-ray diffraction data using CCDC-Mercury software package[1]). X-ray powder diffraction patterns were recorded on an APEX II DUO CCD diffractometer using a IμS CuKα X-ray source. Manually separated crystals or bulk samples were always kept in DMF. They were ground under solvent to produce micro-crystalline samples and transferred with a pipette into capillaries which were filled 1-2 cm in height. The capillaries were mounted and centered on a goniometer head on a Bruker APEX II diffractometer for data collection. The data were collected upon 360° ϕ rotational frames at 2Θ values of 10° and 20°, with exposure times of 10 or 20 minutes per frame at a detector distance of 120 mm. Overlapping sections of data were combined and the data was processed using the Bruker APEX2 routine XRD-Eval subprogram[2].

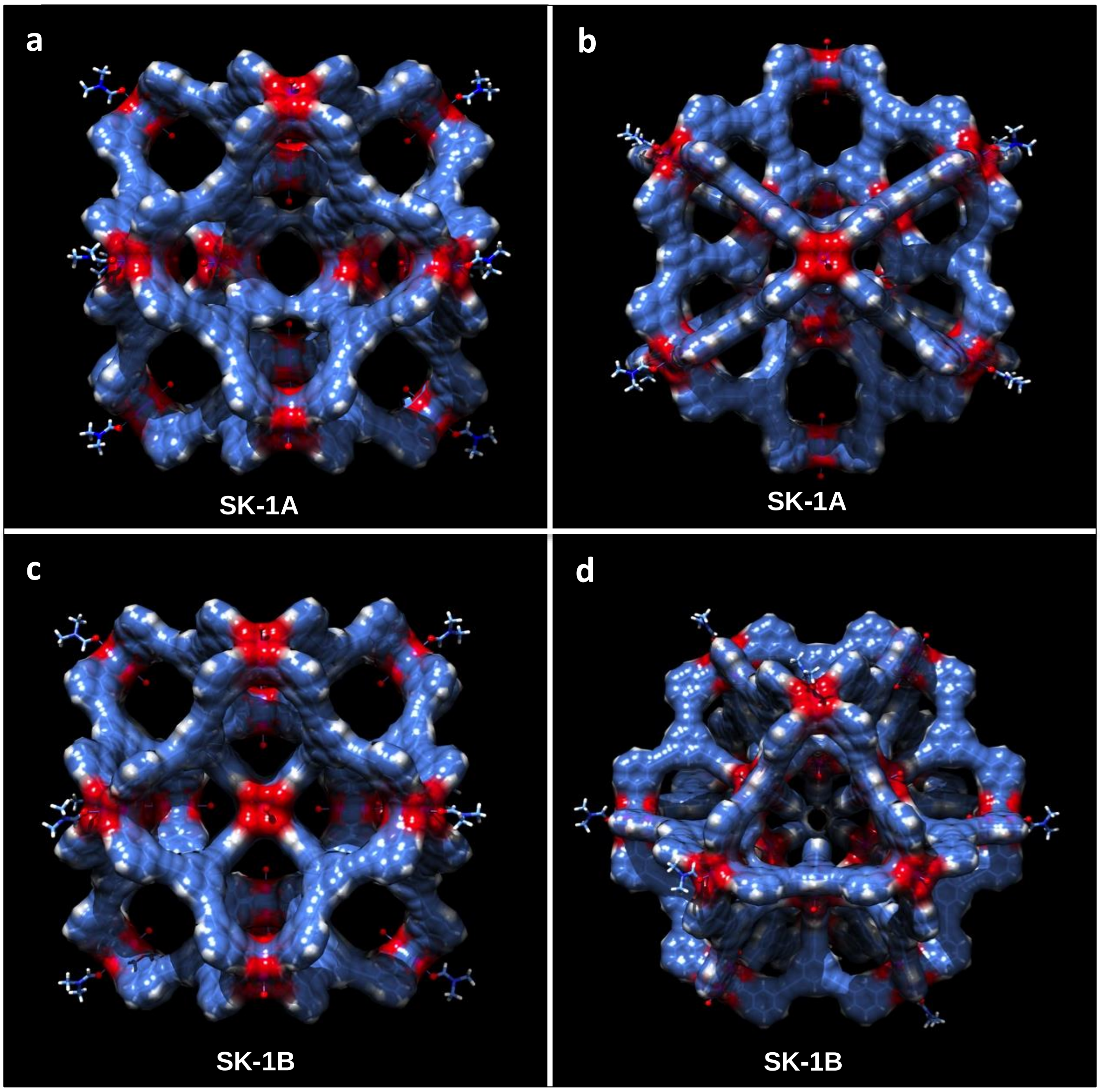

Supplementary Figure 2 | Space-filling representations. Images highlight the square and triangular openings in **SK-1**.

a

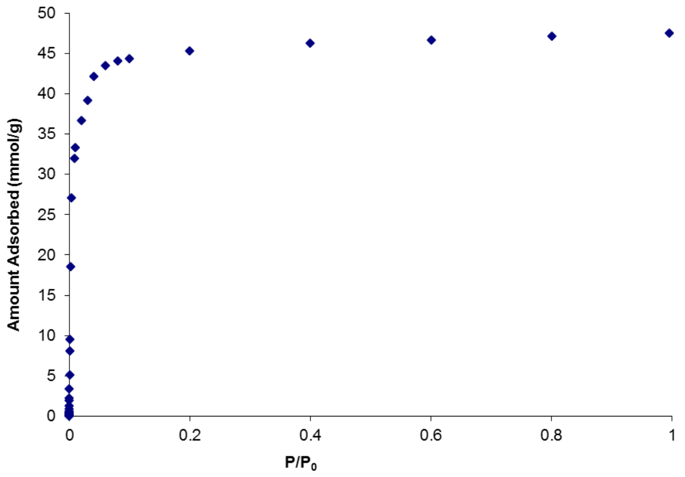

b

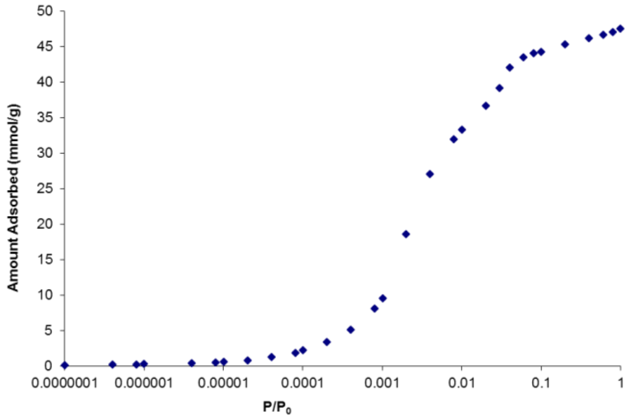

c

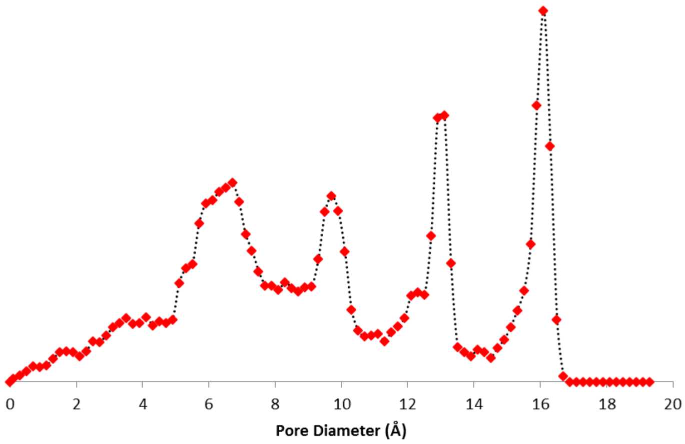

Supplementary Figure 3 | Theoretical calculation of the BET surface area and pore-size distribution of SK-1A. a & b Simulated N_2 adsorption isotherm at 77 K. Surface Area (BET) = 4126 m^2/g. He Pore Volume: 1.63 cm^3/g; **c** Simulated pore size distribution of **SK-1A**.

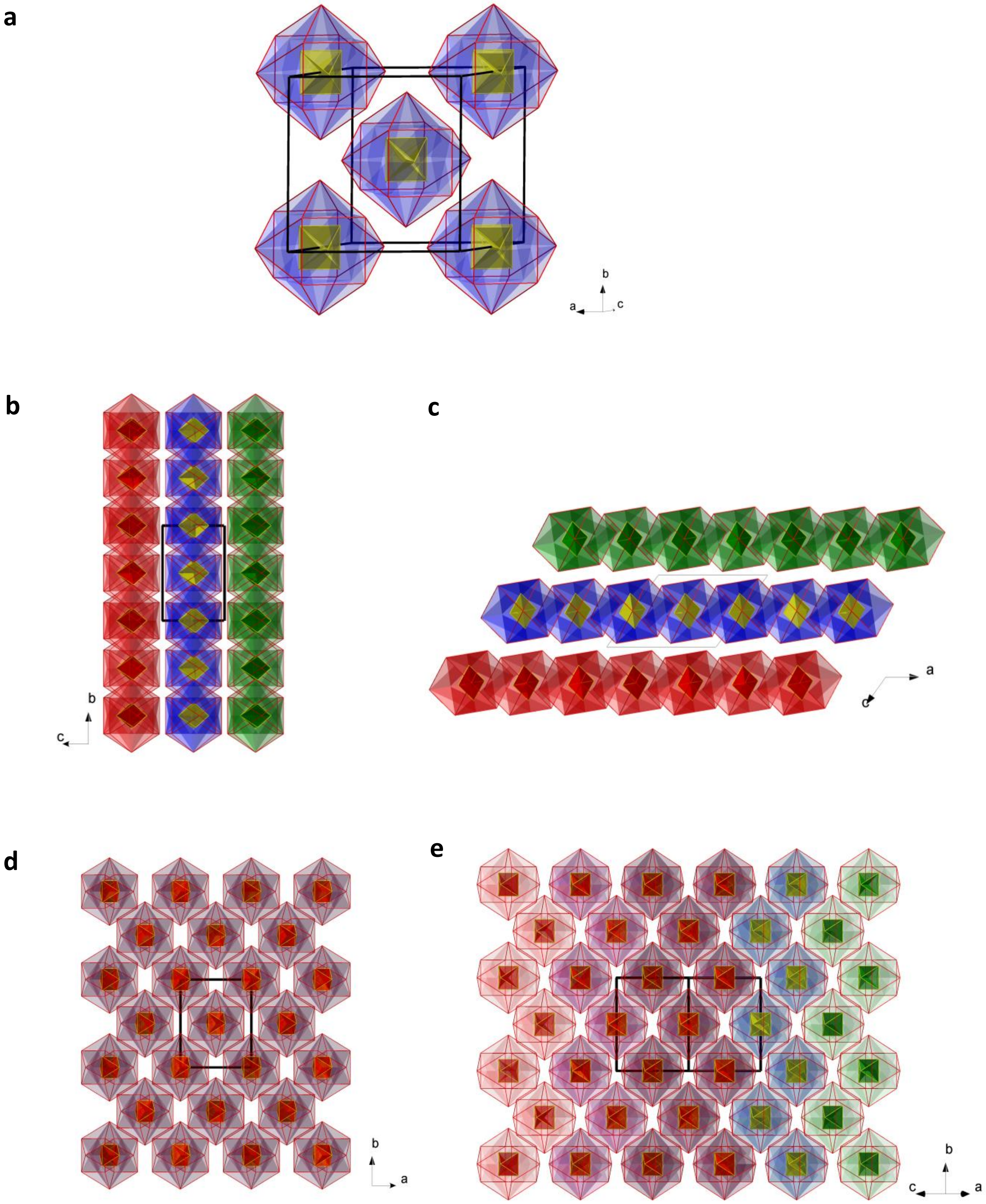

Supplementary Figure 4 | Packing diagrams of SK-1. Simplified representations of the cuboctahedral species; disordered atom positions are omitted for clarity. **a** Unit cell of **SK-1**; **b** & **c** Views in the direction of the crystallographic *a*- and *b*-axes, respectively; **d** & **e** Views in the direction of the crystallographic *c*- and [101]- directions.

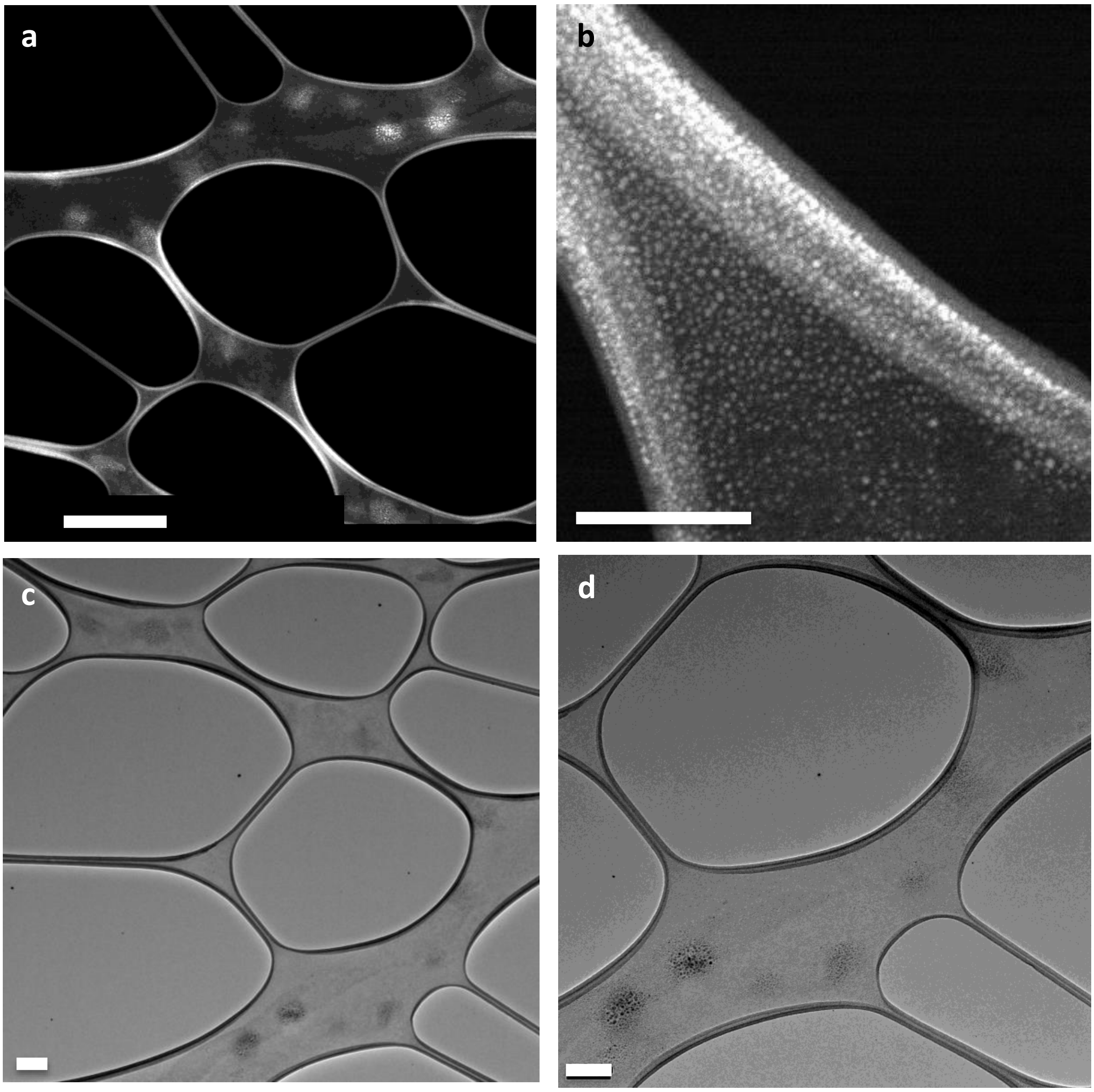

Supplementary Figure 5 | **TEM study.** Overview TEM images of **SK-1**/PPP/$CHCl_3$ samples that were drop-casted on carbon-coated TEM grids (**a** & **b** STEM mode; **c** & **d** TEM mode). Acceleration voltage of 80 kV; scale bars equal 1 µm (**a**); 200 nm (**b**-**d**). Note: Transmission Electron Microscopy (TEM) experiments were carried out using a $CHCl_3$ solution (2 mL) containing **SK-1** (*ca.* $1x10^{-3}$ g) and excess of 4-(3-phenylpropyl)pyridine (*ca.* 100 µL). *Ca.* 0.15 mL of the former solution was drop-casted onto a carbon coated TEM grid which was dried overnight. The samples were imaged using a Titan field emission Transmission Electron Microscope.

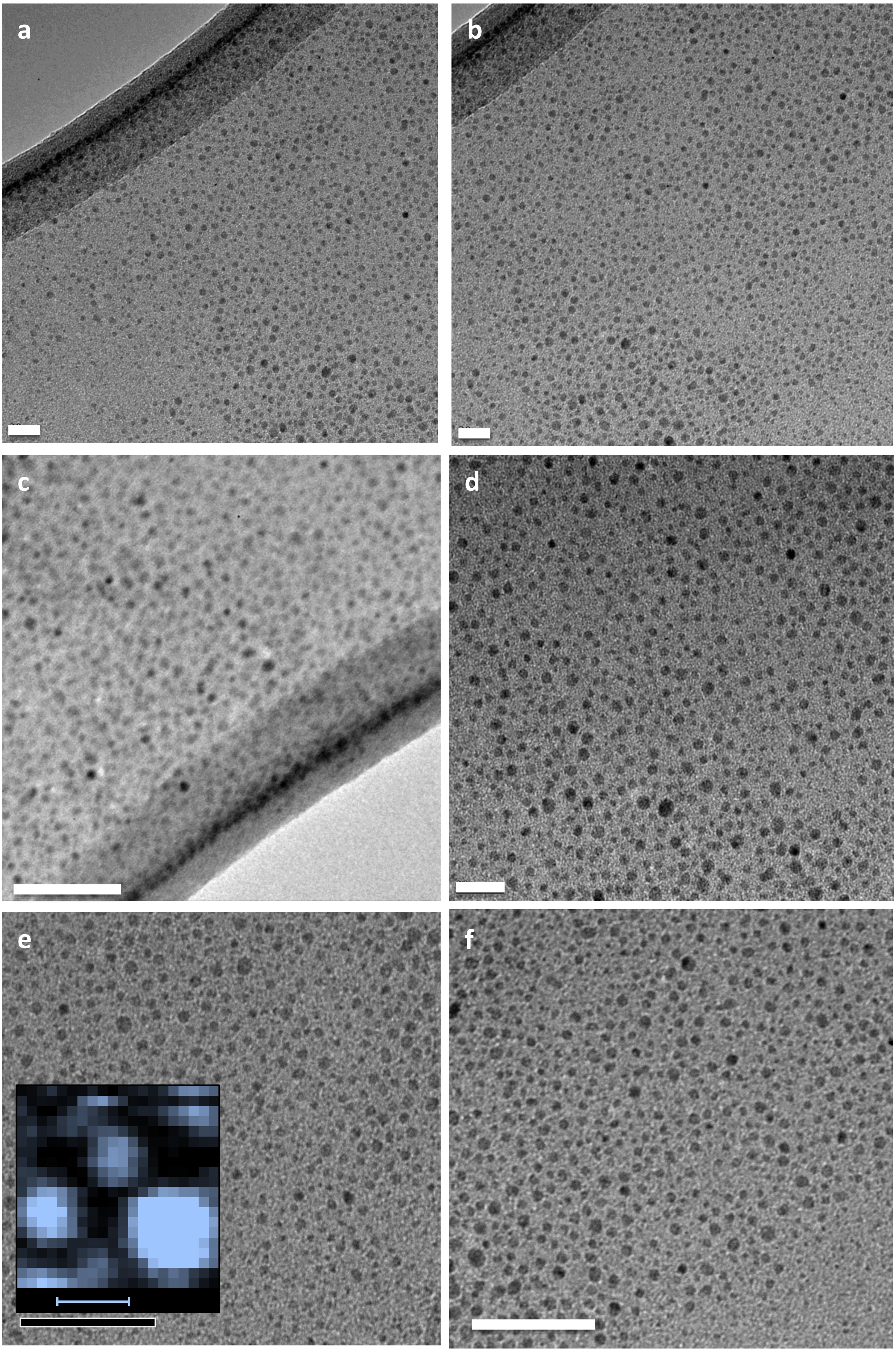

Supplementary Figure 6 | TEM study. TEM images of **SK-1**/PPP/$CHCl_3$ samples that were drop-casted on carbon-coated TEM grids; scale bars equal 20 nm (**a** & **b**); 50 nm (**c**); 20 nm (**d**); 50 nm (**e** & **f**). Inset **e**: EDX mapping of the element Cu; scale bar equals 20 nm. Acceleration voltage of 80 kV.

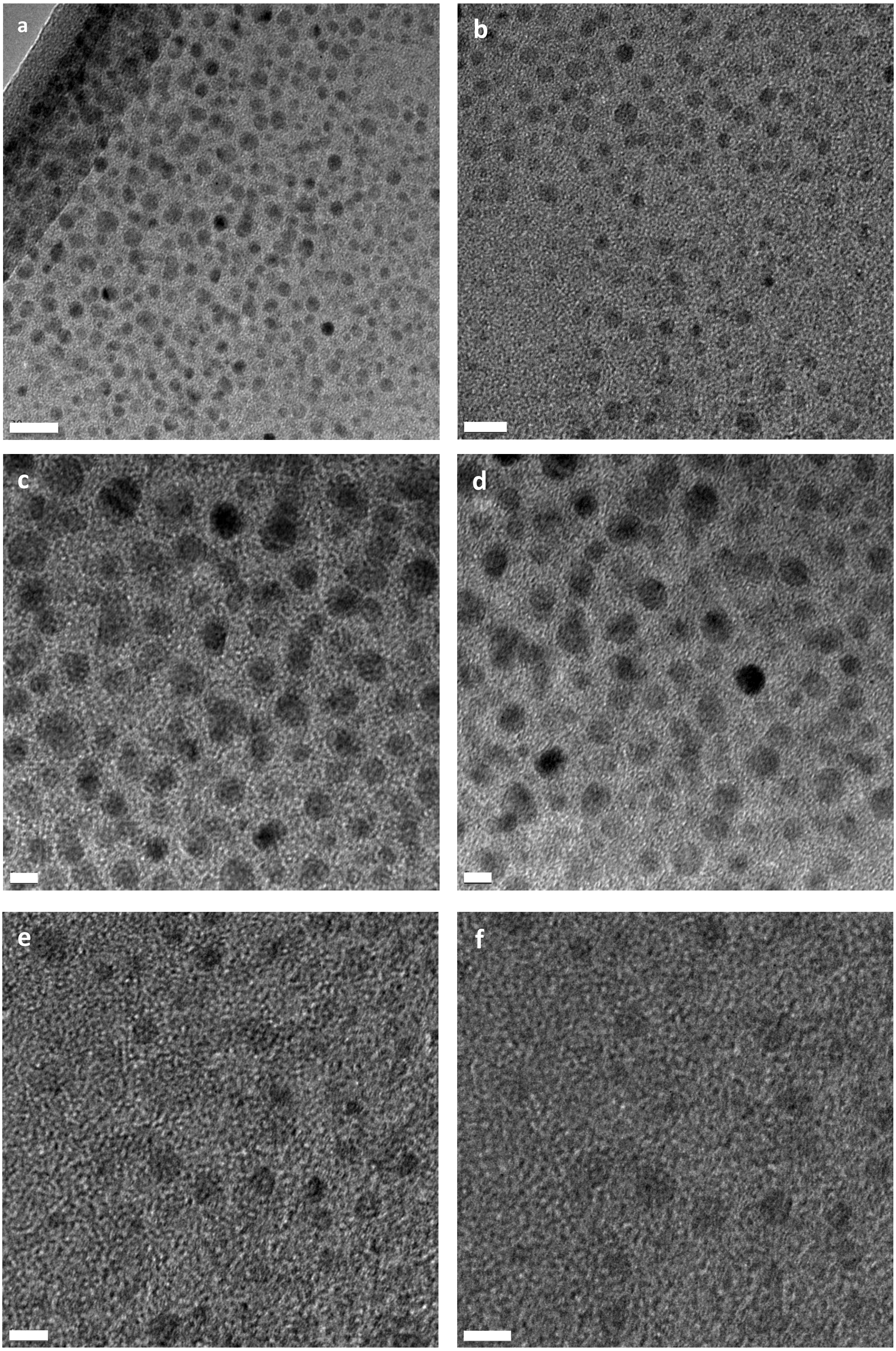

Supplementary Figure 7 | **TEM study.** HR-TEM images of **SK-1**/PPP/$CHCl_3$ samples that were drop-casted on carbon-coated TEM grids. Acceleration voltage of 80 kV; scale bars equal 20 nm (**a**); 10 nm (**b**); 5 nm (**c**-**f**).

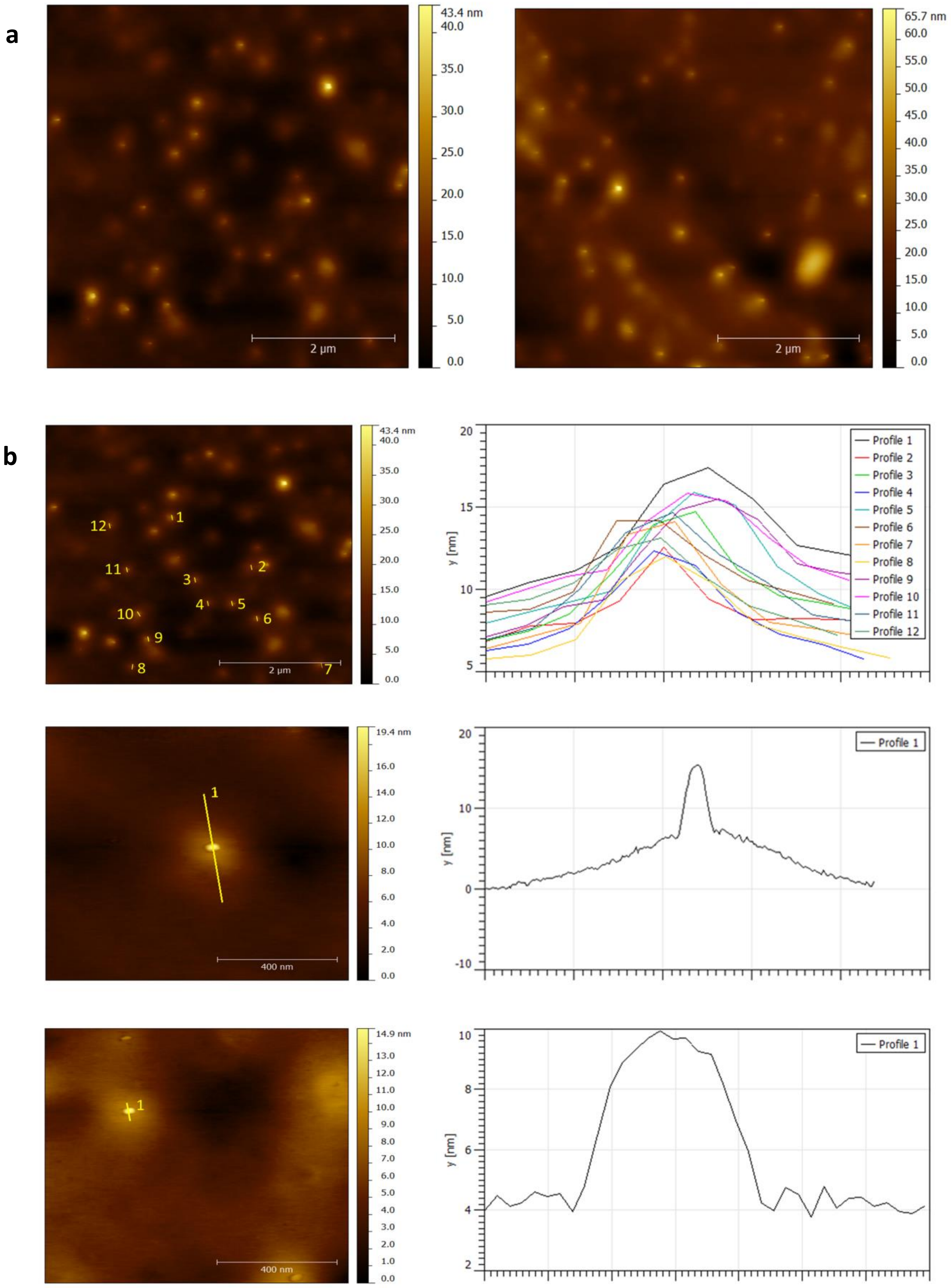

Supplementary Figure 8 | **AFM Studies. a** AFM overview images; **b** AFM/height analysis of individual particles; **SK-1**/PPP/$CHCl_3$ solution dropcasted on HOPG.

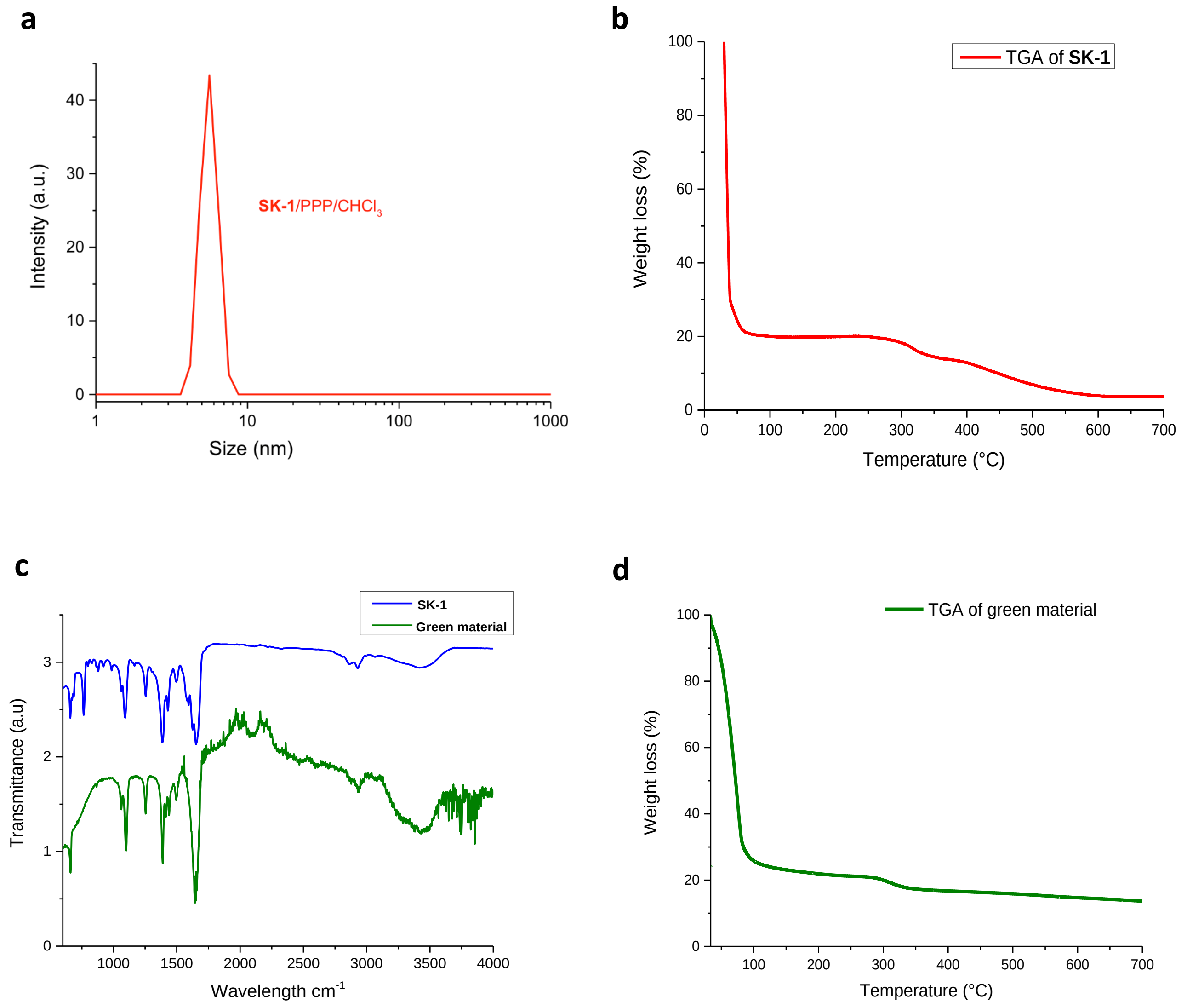

Supplementary Figure 9 | Characterisation of SK-1. a Dynamic light scattering of a saturated solution of **SK-1**. Measurement in $CHCl_3$ using PPP as solubilising co-ligand ([PPP] $7.5x10^{-4}$ M). Corrections were carried out only for the solvent (refractive index and viscosity). Signal centered at 5.62 nm. **b** Thermogravimetric analysis of **SK-1**. TGA measurements were carried out using a Perkin-Elmer Pyris-1 analyser instrument. Crystals were removed from their DMF storage solutions and placed briefly onto filter paper to remove excess solvent prior to their addition to the TGA crucible. The measurements were carried out under N_2 gas flow (20 mL/min) between 30 ^{o}C and 700 ^{o}C. The applied heating rate was 3 ^{o}C per minute. **c** IR spectrum of the co-precipitating green material that forms with **SK-1**, deposited on a platinum ATR accessory (room temperature measurement). *Note*: This material does not dissolve in the presence of PPP indicative of different molecular or 3-dimensional structures present in the material. **d** Thermogravimetric analysis of the co-precipitating green material that forms with **SK-1**; N_2 gas flow (20 mL/min). Sample preparation as for **SK-1**.

a

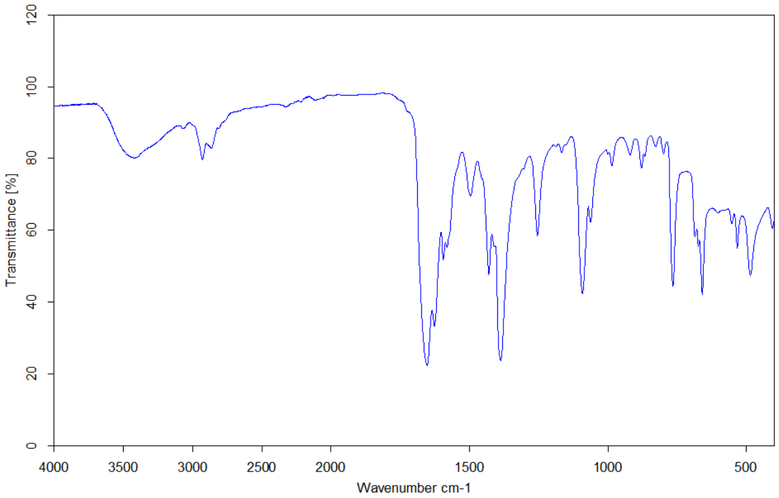

b

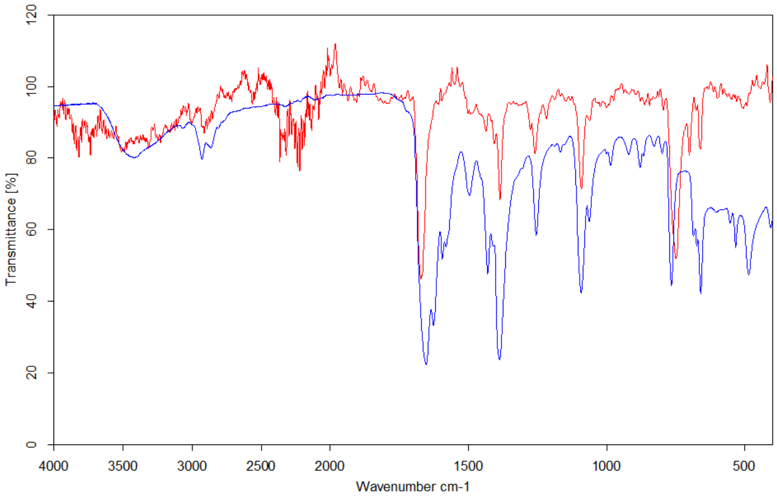

c

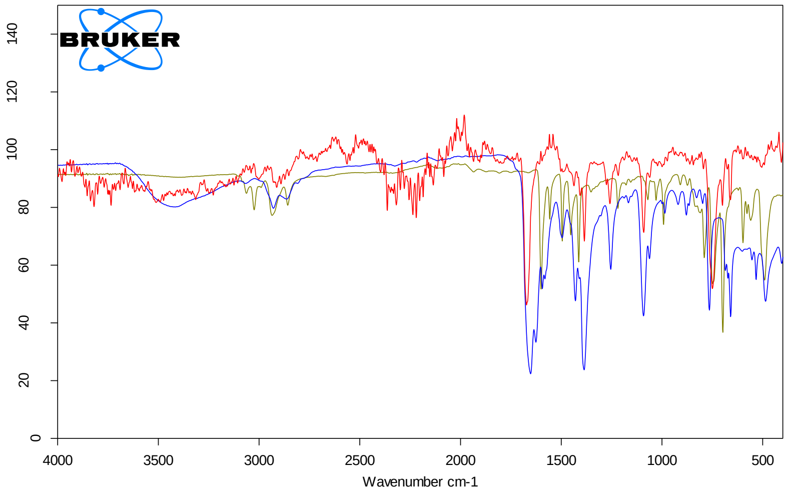

Supplementary Figure 10 | IR Study. a IR spectrum of a crystalline sample of **SK-1. b** Comparison of IR spectra of a crystalline sample of **SK-1** (blue) and of **SK-1**/PPP/$CHCl_3$ deposits (red, solution deposited on a platinum ATR accessory). Signals in the spectrum of **SK-1**/PPP/$CHCl_3$ arising from excess PPP were subtracted by a background measurement. **c** Comparison of IR spectra of a crystalline sample of **SK-1** (blue) and of **SK-1**/PPP/$CHCl_3$ deposits (red) and PPP (green). Signals in the spectrum of **SK-1**/PPP/$CHCl_3$ arising from excess PPP were subtracted by a background measurement. IR spectra were measured on a Bruker TENSOR II FTIR Spectrometer with a platinum ATR accessory.

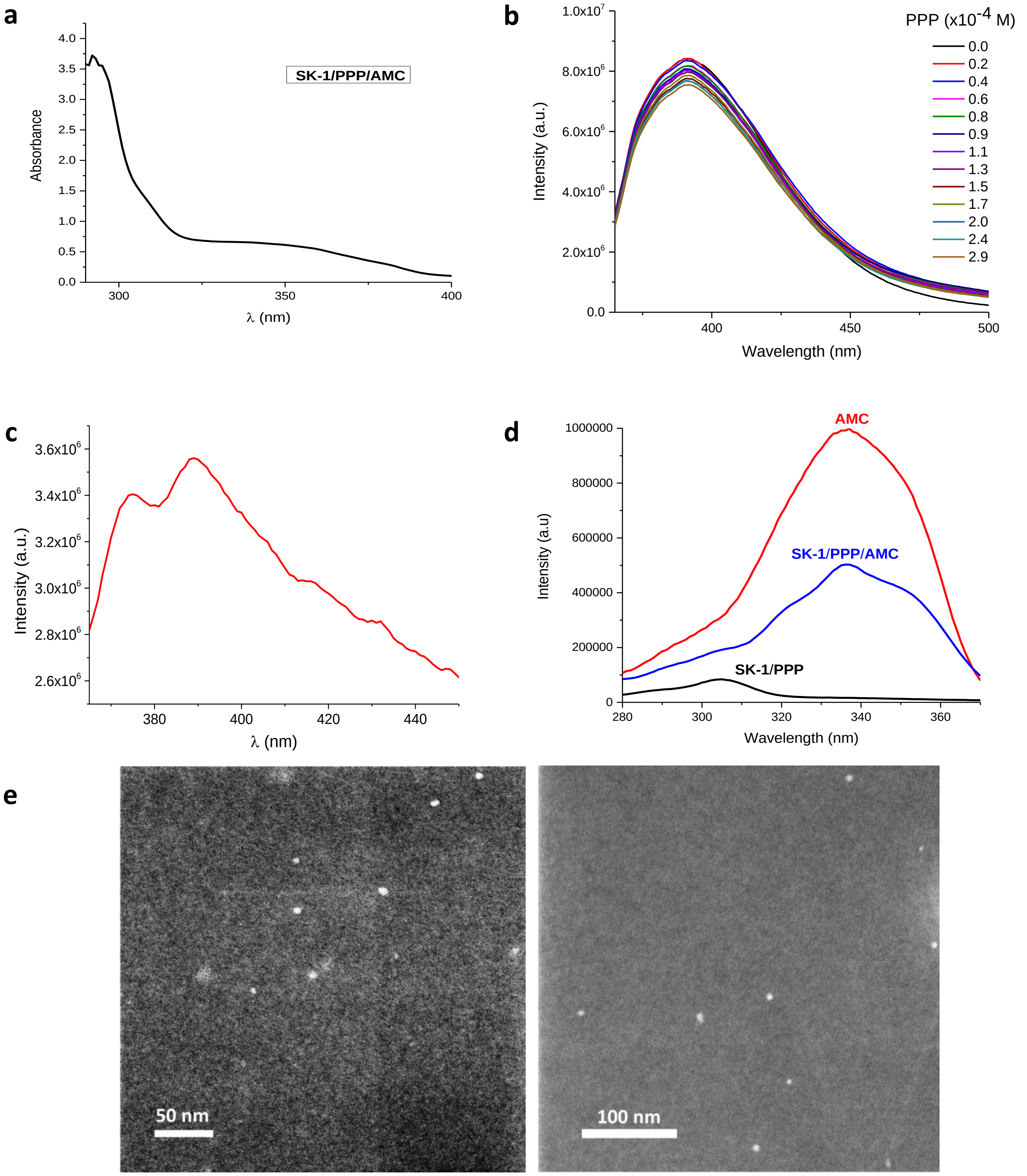

Supplementary Figure 11 | Guest interactions. a UV-vis spectrum of **SK-1** (2 x 10^{-7} M)/PPP (7.5 x 10^{-6} M)/AMC (1 X 10^{-7} M) in $CHCl_3$. **b** Fluorescence control titration when PPP in $CHCl_3$ is added to an AMC solution in $CHCl_3$ (1x10^{-7} M, λ_{ex} = 338 nm). **c** Fluorescence spectrum of **SK-1**/PPP in $CHCl_3$ ([**SK-1**]~2x10^{-5} M, [PPP]~7.6x10^{-4} M, λ_{ex} = 338 nm). **d** Excitation spectra of AMC in $CHCl_3$ (1x10^{-7} M, λ_{em} = 391 nm, red), **SK-1**/PPP/AMC in $CHCl_3$ ([**SK-1**]~ 8x10^{-8} M, [PPP]~3x10^{-6} M, [AMC]~4x10^{-9} M, λ_{em} = 391 nm blue), **SK-1**/PPP ([**SK-1**]~2x10^{-7} M, [PPP]~7.5x10^{-6} M, λ_{em} = 387 nm, black). **e** Scanning transmission electron microscopy (STEM) images of **SK-1**/PPP/AMC/$CHCl_3$ ([**SK-1**]~8x10^{-8} M, [PPP]~3x10^{-6} M, [AMC]~4x10^{-9} M) samples that were drop-casted on carbon-coated TEM grids (binding study involving the addition of **SK-1**/PPP to an AMC/$CHCl_3$ solution); acceleration voltage of 300 kV. Low-concentrated samples that approximately match those used in the photophysical studies.

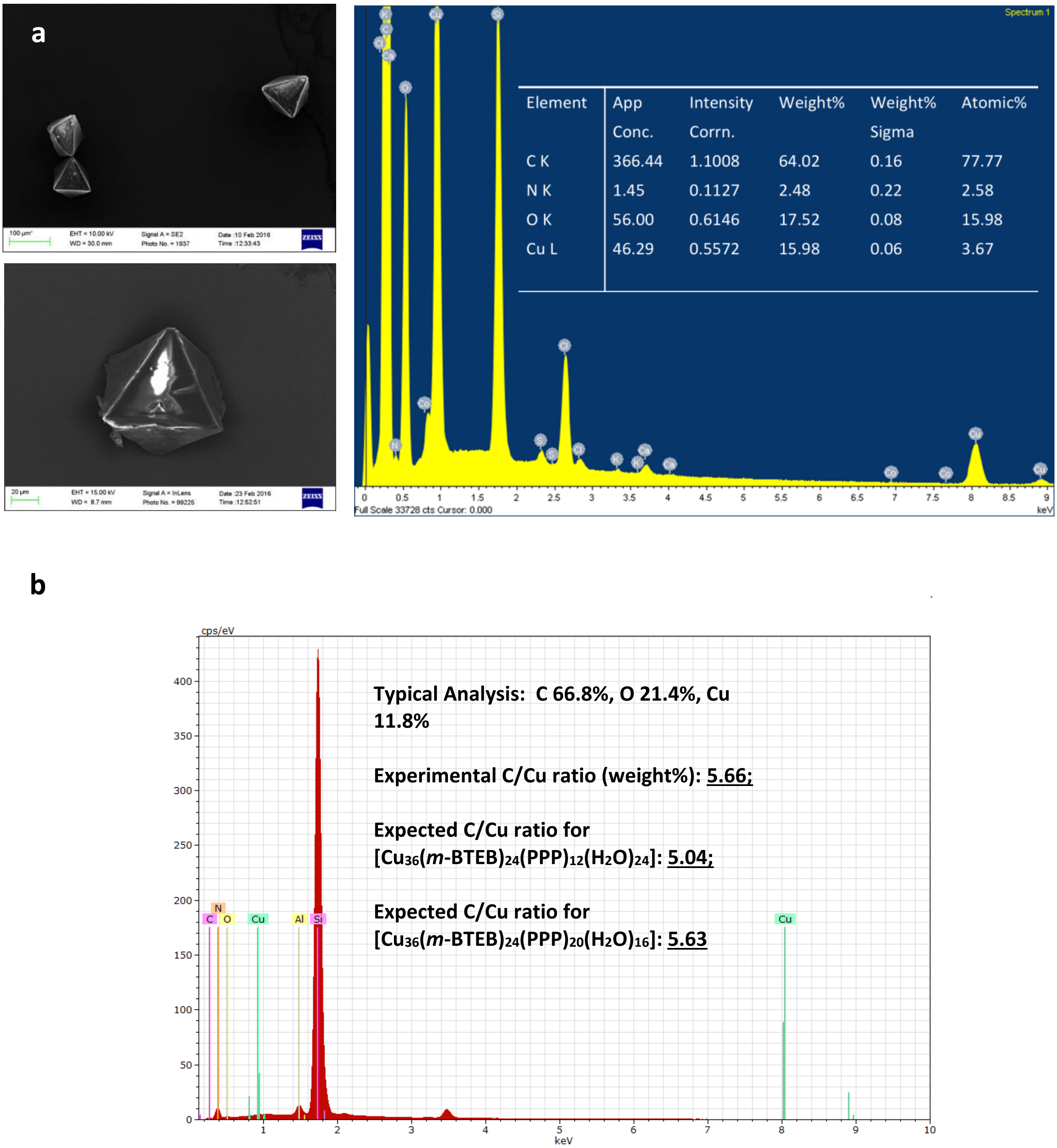

Supplementary Figure 12 | Energy Dispersive X-Ray (EDX) analysis. a EDX spectroscopy of crystals of **SK-1**. Sample stored in $CHCl_3$, dried in air and deposited on a Si/SiO_2 surface. Composition expected for [Cu_{36}(*m*-BTEB)$_{24}$(H_2O)$_8$(DMF)$_{28}$]: C 65.4%, N 2.4%, O 17.9% and Cu 14.2%. **b** EDX spectroscopy of **SK-1**/PPP/$CHCl_3$ samples that were drop-casted on Si_4N_3 TEM grids. Analysis was carried out using solutions that were also used for the TEM sample preparation. For this purposes the dissolved molecules were deposited on Si_4N_3 surfaces or TEM grids.

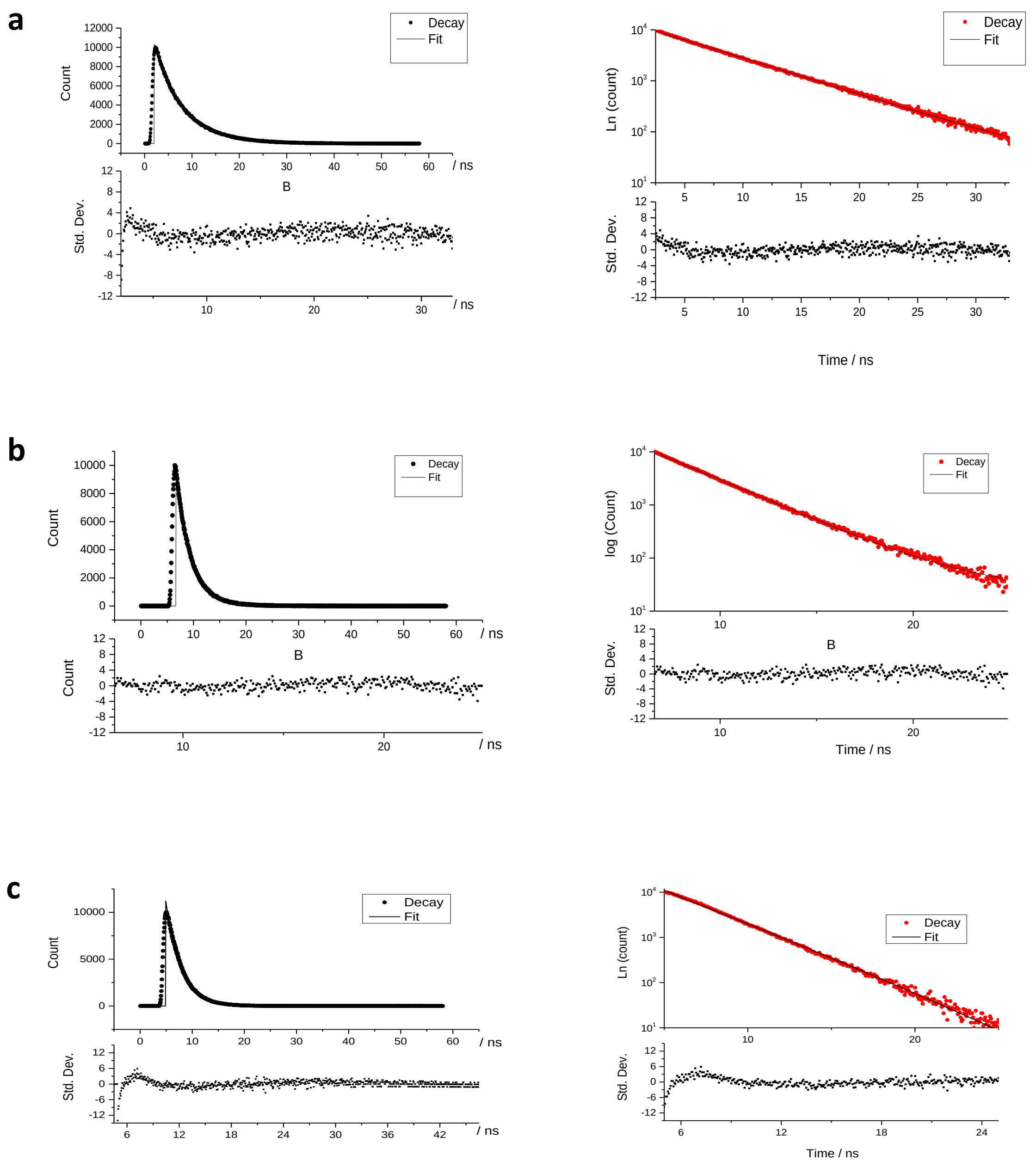

Supplementary Figure 13 | Photophysical Studies. a Lifetime measurement of AMC in $CHCl_3$ (1x10^{-7} M, λ_{ex} = 338 nm, λ_{em} = 391 nm). τ = 2.90 ns (mono-exponential fit). **b** Lifetime measurement of **SK-1**/PPP in $CHCl_3$ ([**SK-1**]~2x10^{-7} M, [PPP]~7.5x10^{-6} M, λ_{ex} = 338 nm , λ_{em} = 391 nm). τ = 6.01 ns (mono-exponential fit). **c** Lifetime measurement of **SK-1**/PPP/AMC in $CHCl_3$ ([**SK-1**]~7x10^{-9} M, [PPP]~7.5x10^{-6} M, [**AMC**] ~1x10^{-7} M, λ_{ex} = 338 nm , λ_{em} = 391 nm) . τ = 2.87 ns (mono-exponential fit).

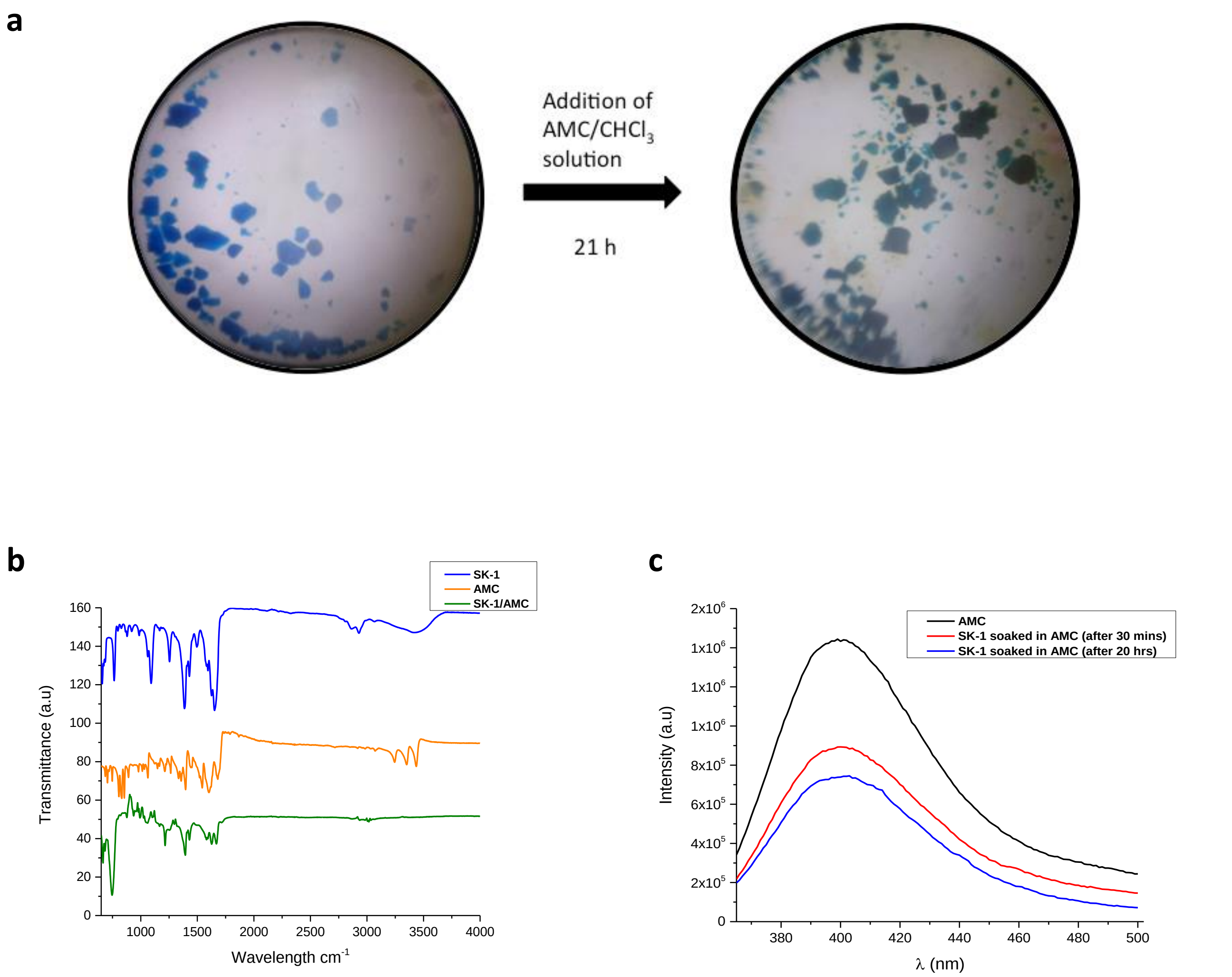

Supplementary Figure 14 | AMC incorporation into crystals of SK-1. **a** Images of crystals of **SK-1** that were dispersed in a solution of AMC in $CHCl_3$ (*ca.* 5 x 10^{-9} M) highlighting the observed colour change; **b** IR spectra of a crystalline sample of **SK-1** (blue) and of AMC (orange) and crystals of **SK-1** that were dispersed in a diluted AMC solution in $CHCl_3$ for *ca.* 16h (green); **c** Fluorescence spectra when a few individual crystals of **SK-1** were added to the solution of AMC in $CHCl_3$ (*ca.* 5 x 10^{-9} M).

a

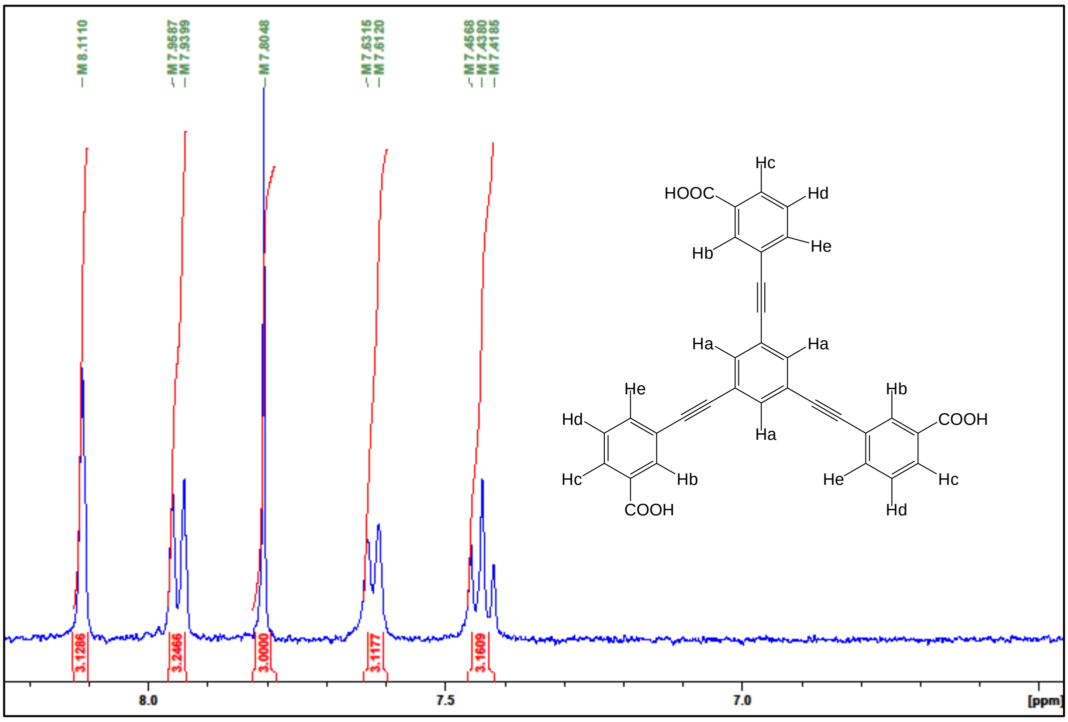

b

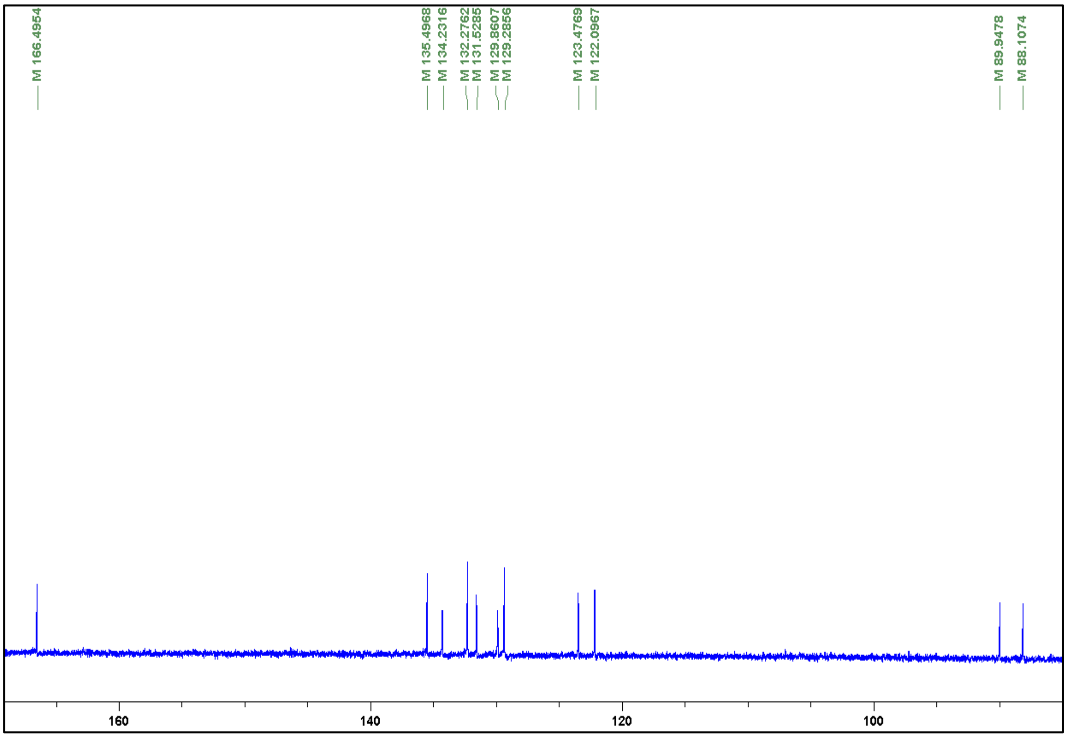

Supplementary Figure 15 | NMR spectra of *m*-BTEB. a ^{1}H-NMR spectrum in DMSO-d^{6}**; b** ^{13}C spectrum in in DMSO-d^{6}.

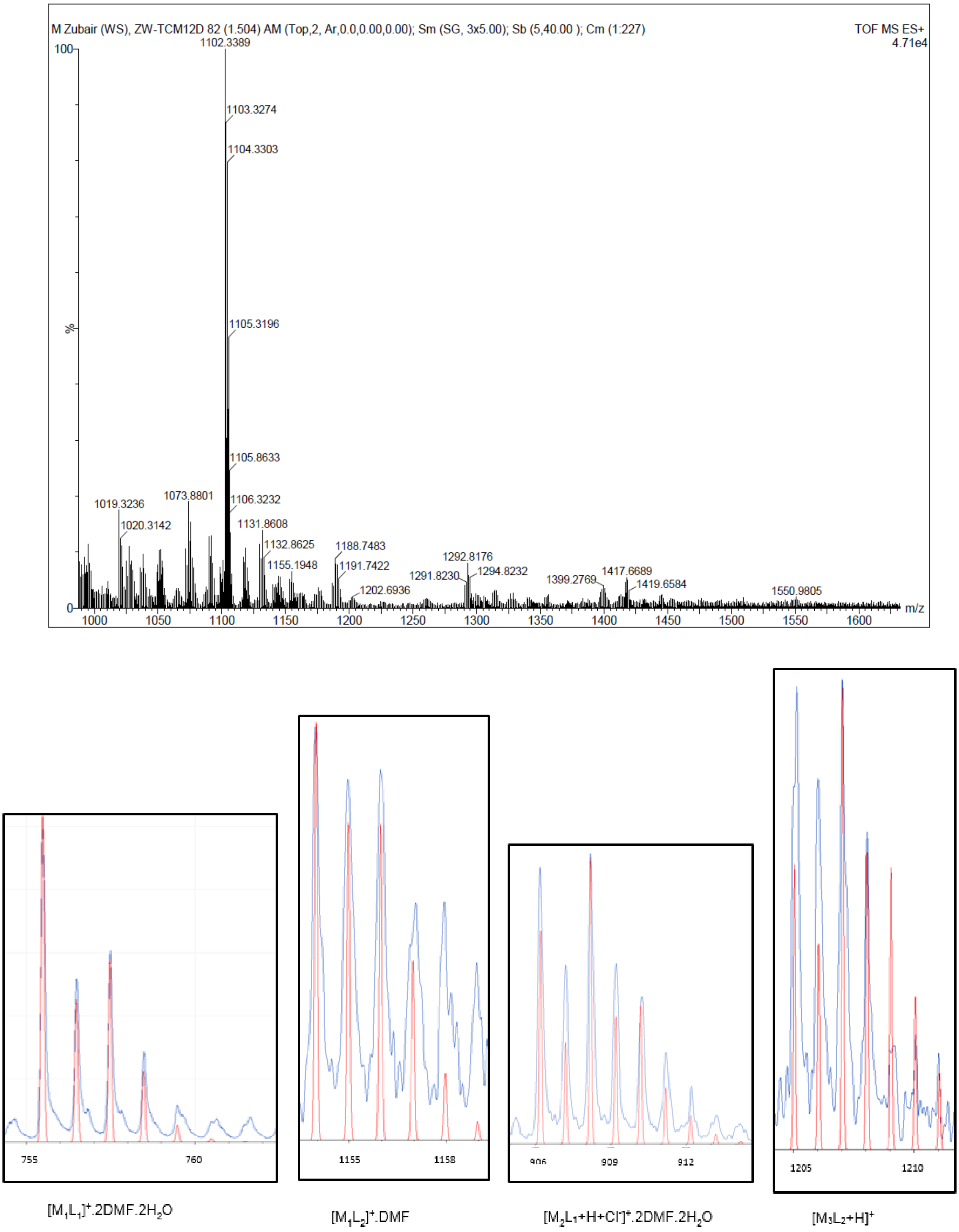

Supplementary Figure 16 | ESI-mass analysis. Spectra of a **SK-1**/PPP solution in $CHCl_3$ highlight the fragmentation of the cage structure under the applied conditions (ESI^+; cone voltage 30 V).

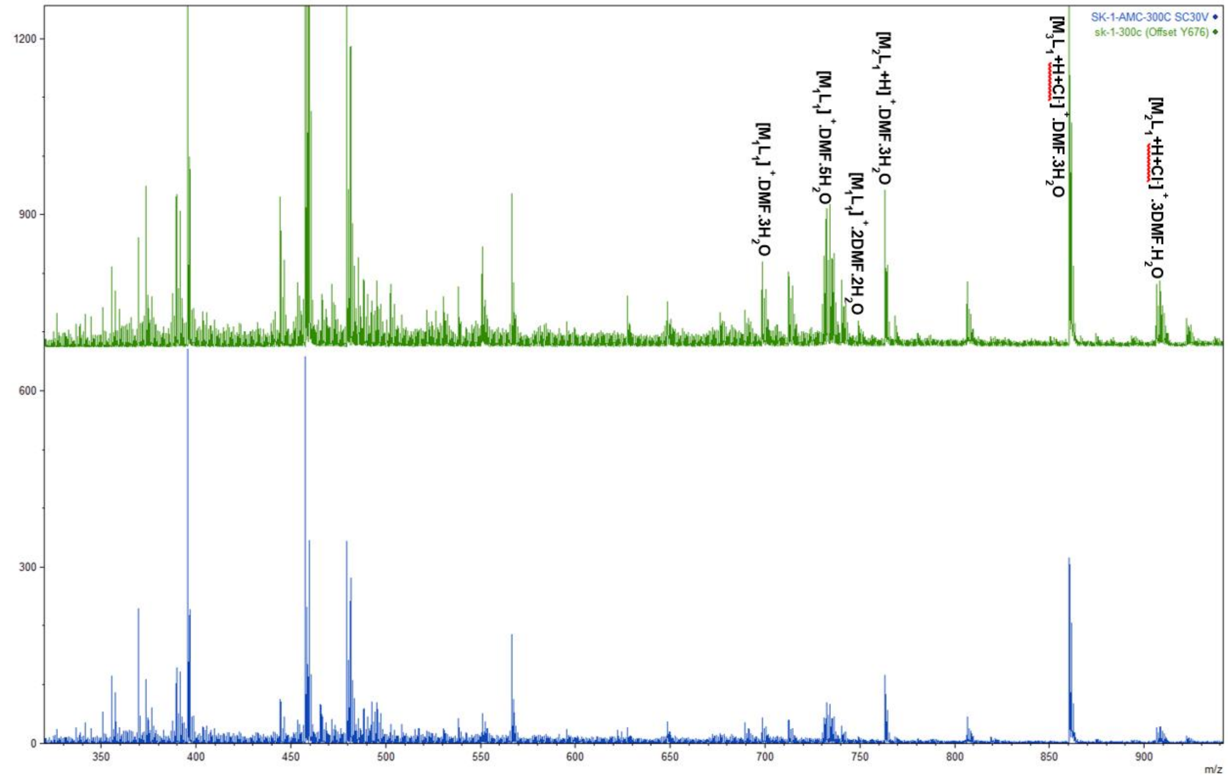

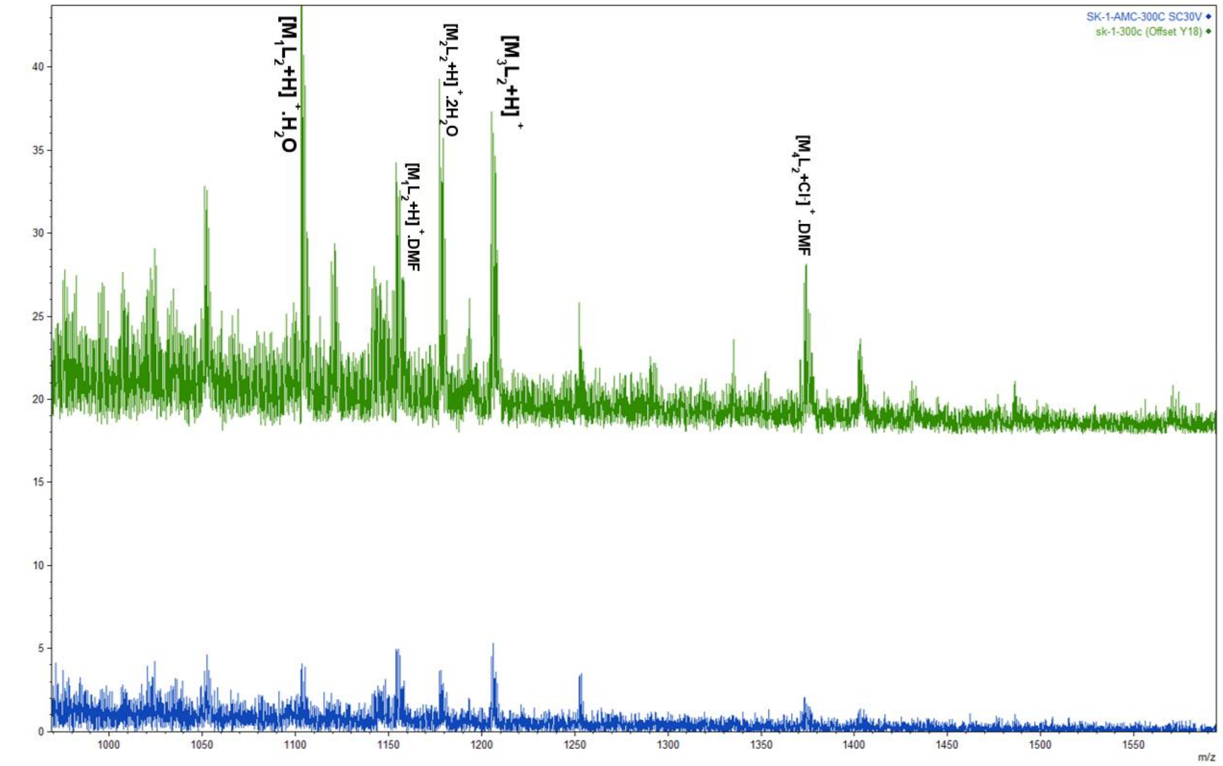

Supplementary Figure 17 | ESI-mass analysis. The spectrum of **SK-1**/PPP/AMC (blue) in $CHCl_3$ reveals comparable fragmentation patterns as for the **SK-1**/PPP (green) in $CHCl_3$.

Supplementary Table 1 | Crystallographic data for **SK-1**.

**Identification code**	sk1_sq	
**Empirical formula**	$C_{816} H_{472} Cu_{36} N_8O_{180}$	
**Formula weight**	15555.43	
**Temperature**	215(2) K	
**Wavelength**	1.54178 Å	
**Crystal system**	Monoclinic	
**Space group**	*C*2/*m*	
**Unit cell dimensions**	*a* = 54.9305(18) Å	α = 90°.
	b = 55.3715(18) Å	β = 125.723(2)°.
	c = 44.3760(16) Å	γ = 90°.
**Volume**	109578(7) $Å^3$	
Z	2	
**Density (calculated)**	0.471 Mg/m^3	
**Absorption coefficient**	0.596 mm^{-1}	
**F(000)**	15816	
**Crystal size**	0.48 x 0.2 x 0.2 mm^3	
**Theta range for data collection**	1.272 to 50.624°.	
**Index ranges**	-55≤h≤54, -55≤k≤55, -41≤l≤43	
**Reflections collected**	433251	
**Independent reflections**	58046 [R(int) = 0.0992]	
**Completeness to theta = 50.624°**	99.0%	
**Absorption correction**	Semi-empirical from equivalents	
**Max. and min. transmission**	0.7500 and 0.3118	
**Refinement method**	Full-matrix least-squares on F^2	
**Data / restraints / parameters**	58046 / 2663 / 2719	
**Goodness-of-fit on F^2**	1.339	
**Final R indices [I>2σ(I)]**	R1 = 0.1280, wR2 = 0.3635	
**R indices (all data)**	R1 = 0.1741, wR2 = 0.3915	
**Largest diff. peak and hole**	0.934 and -0.688e.$Å^{-3}$	
**Total Potential Solvent Accessible Void Vol . ($Å^3$) per unit cell, (%)**	77291 Å; *ca.* 70.5	
**CCDC No.**	1491341	

Supplementary Notes

Supplementary Note 1. Single crystal X-ray data and refinement details for SK-1

Single crystals of **SK-1** were mounted in a capillary containing a small quantity of DMF. Data were collected on a Bruker APEX II DUO CCD diffractometer equipped with a IμS CuKα microfocus tube (wavelength of 1.54184 Å). The single crystal was cooled to 215(2) K, using an Oxford Cobra Cryostream low-temperature device. The diffraction frames were collected to a resolution of 1 Å, integrated and processed using the Bruker APEX2[2] software package. The data were corrected for absorption effects using the multi-scan method (SADABS).[3] The space group was determined using XPREP[4], and the structure was solved by Intrinsic Phasing (XT)[5] and refined using the XL in OLEX2.[6, 7]

The {Cu_2} SBUs in the inside of the sphere are disordered over 18 different positions, of which 6 locate under the square faces and 12 locate under the vertices of the outer cuboctahedron; each inner phenyl ring of the *m*-BTEB ligand adopts three different orientations. The internal ligands were located or refined using restrains/constrains due to the partial occupancies, e.g. benzene rings were constrained as regular hexagons (bond lengths of 1.39 Å) keeping the carboxylate functionalities within the planes of the aromatic ring moieties.-Restraints (DFIX, SIMU, ISOR) were used in the model for some Cu-O or O-O separations and C-C distances. Constraints (EADP) were applied to some oxygen atoms. One outer ligand was disordered - C4R-C10R and C4s-C10s and was modelled in two positions with *ca.* 45:55% occupancy.

In the asymmetric unit, there are six inner dinuclear {Cu_2} paddle-wheel units, in which two locate under the square faces and four locate under the vertices of the outer cuboctahedral structure. The 18 inner {Cu_2} units that result due to symmetry operations can be assigned to two structures of **SK1-A** and **SK1-B**, which can be regarded as disordered over four positions. **SK1-A** contains 2 inner units under the square faces and four under the outer vertices. This molecular entity is disordered over three positions. **SK1-B** contains 6 inner {Cu_2} units that all locate under the square faces, and these units are 'shared' with the three **SK1-A** sub-structures. With this in mind, the occupancies of these inner units were refined freely, except the units belonging to the same disorder were assumed to have the same occupancies. The results indicate that **SK1-A** dominates, while **SK1-B** may exist in low quantity. The reported results are based on the model, in which the total occupancy parameters of the disordered atoms add up to 1.

Hydrogen atoms were added (except for those of some C atoms which are shared by different disorder modes) in idealised geometries and allowed to ride on their carrier atoms with an isotropic displacement parameter related to the equivalent displacement parameter of their carrier atoms. Hydrogen atoms of coordinating H_2O molecules are highly disordered and were also omitted. The structure contains huge solvent accessible void volumes in which solvent molecules could not be located (see BET calculations). To account for this, the Platon-Squeeze routine[8] was used to calculate the void volume and re-generate the reflection file by excluding the diffraction contributions of these unlocated solvent molecules in void spaces between the molecules. The R1 value changed from 20.0% to 12.8% after applying the Platon-squeeze routine. The final results are based on the new reflection data. The total potential solvent accessible void volume accounts for 77291 Å^3 per unit cell (*ca.* 70.5% of the unit cell volume). The sum formula included in the CIF file reflects the ideal formula of **SK-1** and excludes any solvent molecules in the voids. Crystal data and numerical details for the data collection and refinement are given in Table S1. The final, fractional atomic coordinates, equivalent displacement parameters and anisotropic displacement parameters for the non-hydrogen atoms are given in the CIF. The programs Diamond 3[9] and Chimera[10] were used to prepare the images of the structure.

Supplementary Note 2. BET Simulation details and pore-size distribution

The adsorption of N_2 at 77 K was simulated using the grand canonical Monte Carlo simulations implemented in the MuSiC software package[11] and using translation, rotation and energy-biased insertion and deletion moves. All simulations were allowed at least 8 x 10^6 equilibration steps, followed by 12 x 10^6 production steps for each pressure point. The system treated as rigid, with MOP atoms kept fixed at their crystallographic positions. Lennard-Jones parameters for the framework atoms were taken from the DREIDING force field[12] with the exception of Cu, for which UFF parameters[13] were used. Nitrogen was simulated as a rigid molecule using the TraPPE mode[14], incorporating both Lennard-Jones parameters and partial charges. Previous work has shown that nitrogen-MOF electrostatic contributions play only a minor role in nitrogen adsorption[15] and, as such, only the electrostatic interactions between nitrogen molecules were included in our simulations. The BET surface area were calculated following the consistency criteria of Rouquerol and co-workers[16] using a pressure range of $0.004 < P/P_0 < 0.06$. The data was simulated using a cif-file of **SK-1** in which disordered atom positions were deleted to represent **SK-1B**. All constitutional solvent molecules were removed whilst coordinated solvent molecules (DMF & H_2O) were retained.

The pore size distribution (PSD) was calculated following the method of Gelb and Gubbins[17], in which the largest diameter spheres which may be successfully inserted into the structure without overlap with any of the framework atoms are recorded. The helium pore volume was determined using the Poreblazer tool set[18] using standard helium Lennard-Jones parameters (σ = 2.51 Å, ε/k_B = 10.22 K)[19].

Supplementary Methods

Synthesis of 3,3′,3′′-(benzene-1,3,5-triyltris(ethyne-2,1-diyl)tribenzoic acid (*m*-BTEB)

3,3′,3′′-(Benzene-1,3,5-triyltris(ethyne-2,1-diyl)tribenzoic acid (*m*-BTEB) was synthesised in a 4-step synthesis using a modified, previously described synthetic procedure for the isomeric compound, 4,4′,4′′-(benzene-1,3,5-triyltris(ethyne-2,1-diyl)tribenzoic acid[20].

2,2',2''-(Benzene-1,3,5-triyltris(ethyne-2,1-diyl))tris(propan-2-ol) (I)

2,2',2''-(Benzene-1,3,5-triyltris(ethyne-2,1-diyl))tris(propan-2-ol) (**I**) was synthesised under N_2 atmosphere by stirring 1,3,5-tribromobenzene (10.098 g, 0.032 mol), $Pd(PPh_3)_2Cl_2$ (0.3 g, 0.427 mmol), PPh_3 (0.3 g, 1.143 mmol), 2-methyl-3-butyn-2-ol (16.190 g, 0.192 mol) and triethylamine (200 mL) in a round bottom flask at 20 °C. CuI (0.2 g, 1.050 mmol) was added and the temperature was increased to 80 °C and kept for 24 hours. The resulting precipitate was filtered off and the solution was evaporated to dryness using a rotary evaporator. The resulting crude product was purified using column chromatography (mobile phase: 75% ethyl acetate; 25% petroleum ether). A pale yellow solid was obtained. Yield: 7.673 g, 0.0236 mol, 74%. ^{1}H NMR (400 MHz, DMSO-d, δ_H) 7.39 (3H, s, CH), 5.29 (3H, s, OH), 1.58 (18H, s, CH_3).

1,3,5-Triethynylbenzene (II)

KOH (9.427 g, 0.168 mol) was dissolved in MeOH (20 mL) and added to a stirred solution of **I** (7.570 g, 0.050 mol) in toluene (100 mL). The reaction mixture was stirred for 24 hours before it was neutralised using an aqueous 3 M HCl solution (55 mL). The reaction mixture was concentrated using a rotary evaporator. The crude product was purified using column chromatography (with a mobile: 25% ethyl acetate; 75% CH_2Cl_2). A yellow solid of **II** was obtained. Yield: 3.456 g, 0.023 mol, 46%. ^{1}H NMR (400 MHz, $CDCl_3$-d, δ_H) 7.56 (3H, s, CH), 3.10 (3H, s, CH).

Triethyl-3,3',3''-(benzene-1,3,5-triyltris(ethyne-2,1-diyl))tribenzoate (III)

Triethyl-3,3',3''-(benzene-1,3,5-triyltris(ethyne-2,1-diyl))tribenzoate (**III**) was synthesised under N_2 atmosphere by dissolving ethyl 3-iodobenzoate (7.19 g, 0.026 mol), $Pd(PPh_3)_2Cl_2$ (0.8 g, 0.001 mol), PPh_3 (0.8 g, 0.003 mol), 1,3,5-triethynylbenzene (**II**) (1.185 g, 0.0078 mol) in 50 mL triethylamine at room temperature. Then, CuI (0.75 g, 0.0039 mol) was added to the reaction mixture and the temperature was increased to 35 °C and kept for 40 hours. The mixture was evaporated to dryness and CH_2Cl_2 (15 mL) was added to dissolve the crude product. The insoluble residue was filtered off. The crude product was purified using column chromatography (mobile phase CH_2Cl_2). A white solid of **III** was obtained. Yield: 3.369 g, 0.0056 mol, 72%. ^{1}H-NMR (400MHz, $CDCl_3$-*d*) δH/ppm: 8.22 (3H, s, Ar-H*a*), 8.04 (3H, d, J = 7.89, Ar-H*c*), 7.73-7.70 (6H, m, Ar-H*b,* H*d*), 7.46 (3H, t, J = 7.81, Ar-H*e*), 4.41 (6H, q, J_1 = 7.04 , J_2 = 7.20, CH_2), 1.43 (3H, t, J = 7.18, CH_3); ^{13}C-NMR (400MHz, $CDCl_3$-*d*) δH/ppm: 165.9, 135.7, 134.4, 132.8, 130.9, 129.6, 128.6, 123.8, 123.1, 89.7, 88.4, 61.3, 14.3. HR-MS (ESI): m/z Calculated for $C_{39}H_{31}O_6$ $[M+H]^+$: 595.2121 ; Found: 595.2127.

3,3',3''-(Benzene-1,3,5-triyltris(ethyne-2,1-diyl))tribenzoic acid (*m*-BTEB)

$LiOH.H_2O$ (4.5 g, 0.1072 mol) was dissolved in deionised H_2O (25 mL) and added to a solution of triethyl-3,3',3''-(benzene-1,3,5-triyltris(ethyne-2,1-diyl))tribenzoate (3.2 g, 0.0053 mol) in THF (60 mL). The reaction mixture was stirred for 24 hours before the volume of the solution was reduced using a rotary evaporator. Upon addition of 3 M, aqueous HCl (40 mL), a brown precipitate formed which was filtered off, washed with deionised H_2O (50 mL) and dried in air. The crude brown solid was purified using column chromatography (mobile phase THF). A pale yellow solid of ***m*-BTEB** was obtained. Yield: 2.267 g, 0.0044 mol, 83%; ^{1}H NMR (400MHz, DMSO-d_6) δH/ppm: 8.11 (3H, s, Ar-H*a*), 7.94 (3H, d, J = 7.70, Ar-H*c*), 7.80 (3H, s, Ar-H*b*), 7.62 (3H, d, J = 7.54, Ar-H*e*), 7.43 (3H, t, J = 7.62, Ar-H*d*). ^{13}C NMR (150 MHz, DMSO-d_6) δC/ppm: 166.4, 135.4, 134.2, 132.2, 131.5, 129.8, 129.2, 123.4, 122.0, 89.9, 88.1. HR-MS (ESI): m/z Calculated for $C_{33}H_{17}O_6$ $[M-H]^-$: 509.1025; Found: 509.1027.

Supplementary References

1 Macrae, C. F. et al. Mercury CSD 2.0 - New features for the visualization and Investigation of crystal structures. *J. Appl. Cryst.*, **41**, 466-470, (2008).

2 Bruker. APEX2. (Bruker AXS Inc., Madison, Wisconsin, USA. 2015).

3 Bruker. SADABS. (Bruker AXS Inc., Madison, Wisconsin, USA. 2014).

4 Bruker. XPREP. (Bruker AXS Inc., Madison, Wisconsin, USA. 2014).

5 Sheldrick, G. M. SHELXT – Integrated space-group and crystal-structure determination. *Acta Cryst*. **A71**, 3-8, (2015).

6 Sheldrick, G. M. A short history of SHELX. *Acta Cryst.* **A64**, 112-122, (2008).

7 Dolomanov, O. V. Bourhis, L. J. Gildea, R. J. Howard, J. A. K. Puschmann, H. OLEX2: a complete structure solution, refinement and analysis program. *J. Appl. Cryst.,* **42**, 339-341, (2009).

8 Spek, A. L. PLATON SQUEEZE: a tool for the calculation of the disordered solvent contribution to the calculated structure factors. *Acta Cryst.,* **C71**, 9-19, (2015).

9 Putz, H. Brandenburg, K. Diamond version 3.1c (Crystal and molecular structure visualization crystal impact GbR, Kreuzherrenstr. 102, 53227 Bonn, Germany).

10 Pettersen, E.F. *et al.* UCSF Chimera - A visualization system for exploratory research and analysis. *J. Comput Chem.,* **25**, 1605-1612, (2004).

11 Gupta, A. Chempath, S. Sanborn, M. J. Clark, L. A. Snurr, R. Q. Object-oriented programming paradigms for molecular modeling. *Mol. Simul.,* **29**, 29-46, (2003).

12 Mayo, S.L. Olafson, B. D. Goddard, W.A. DREIDING: a generic force field for molecular simulations. *J. Phys. Chem.*, **94**, 8897-8909, (1990).

13 Rappe, A. K. Casewit, C. J. Colwell, K. S. Goddard, W. A. Skiff, W. M. UFF, a full periodic table force field for molecular mechanics and molecular dynamics simulations. *J. Am. Chem. Soc.*, **114**, 10024-10035, (1992).

14 Potoff, J. J. Siepmann, J. I. Vapor-liquid equilibria of mixtures containing alkanes, carbon dioxide, and nitrogen. *AIChE J.*, ***47***, 1676-1682, (2001).

15 Walton, K. S. Snurr, R. Q. Applicability of the BET method for determining surface areas of microporous metal-organic frameworks. *J. Am. Chem. Soc.*, **129**, 8552-8556, (2007).

16 Llewellyn, P. L., Rodriquez-Reinoso, F. Rouqerol, J. Seaton, N. Characterization of porous solids VII: proceedings of the 7th international symposium on the characterization of porous solids. (Elsevier , 2006).

17 Gelb, L. D. Gubbins, K. E. Characterization of porous glasses: simulation models, adsorption isotherms, and the brunauer-emmett-teller analysis method. *Langmuir*, **14**, 2097-2111, (1998).

18 Sarkisov, L. Harrison, A. Computational structure characterisation tools in application to ordered and disordered porous materials. *Mol. Simul.*, **37**, 1248-1257, (2011).

19 Reid, R. C. Prausnitz, J. M. Poling, B. E. The properties of gases and liquids. (McGraw-Hill, New York, 1987).

20 Castellano, R. K. and Rebek Jr., J. Formation of discrete, functional assemblies and informational polymers through the hydrogen-bonding preferences of calixarene aryl and sulfonyl tetraureas. *J. Am. Chem. Soc.* **120**, 3657-3663, (1998).
